# Descending Pain Modulation Across Pain States: Circuit Mechanisms, Neurochemical Plasticity, and Translational Perspectives

**DOI:** 10.3390/neurolint18100184

**Published:** 2026-09-26

**Authors:** Armando Almeida, Isaura Tavares

**Affiliations:** 1 Life and Health Sciences Research Institute (ICVS), School of Medicine, University of Minho, Campus of Gualtar, 4710-057 Braga, Portugal; 2 ICVS/3B’s—PT Government Associate Laboratory, 4710-057 Braga, Portugal; 3 ICVS/3B’s—PT Government Associate Laboratory, 4805-017 Guimarães, Portugal; 4 Unit of Experimental Biology, Department of Biomedicine, Faculty of Medicine, University of Porto, 4200-319 Porto, Portugal; isatav@med.up.pt; 5 Institute for Research and Innovation in Health and IBMC (i3S), University of Porto, 4200-135 Porto, Portugal

**Keywords:** descending pain modulation, chronic pain, pain facilitation, pain inhibition, periaqueductal gray, rostral ventromedial medulla, dorsal reticular nucleus, conditioned pain modulation, nociplastic pain, pain chronification

## Abstract

Descending pain modulation is a major determinant of pain processing across physiological and pathological conditions. Although descending pain modulation has long been recognized as bidirectional, capable of both inhibiting and facilitating nociceptive transmission, recent advances now allow it to be examined with far greater anatomical, cellular, neurochemical, and functional precision. Circuit tracing, optogenetic and chemogenetic manipulation, molecular profiling, connectomics, and human neuroimaging have expanded classical brainstem models, revealing a modulatory network in which inhibitory and facilitatory influences are dynamically recruited according to physiological and pathological state. In this narrative review, we revisit descending pain modulation across acute, inflammatory, neuropathic, and nociplastic pain states. We discuss canonical pathways, including the periaqueductal gray–rostral ventromedial medulla axis and locus coeruleus–spinal noradrenergic projections, together with the established dorsal reticular nucleus system and more recently identified descending circuits. We examine how circuit organization, neurochemical diversity, and local and long-range network interactions shape modulatory output across pain conditions. Attention is given to state-dependent plasticity and to the progressive reorganization of descending systems during pain chronification. In this context, reduced inhibitory flexibility and enhanced facilitatory bias emerge as recurrent, but not uniform, features of chronic pain. We also consider how human experimental paradigms, pharmacological approaches, and neuroimaging findings can be interpreted in light of these mechanistic advances, while acknowledging the limits of translating circuit-specific animal findings into integrated human measures. By framing descending pain modulation as a distributed and dynamically reconfigurable network rather than as a unitary analgesic system, this review highlights its relevance to pain phenotyping, treatment stratification, and mechanism-informed therapeutic strategies.

## 1. Introduction

Pain is not a fixed readout of peripheral nociceptive input. It emerges from dynamic interactions between ascending signals and modulatory systems distributed across spinal, brainstem, and forebrain circuits. Among these systems, descending pain modulation occupies a central position because it can either suppress or amplify nociceptive transmission according to context, behavioral relevance, and physiological state.

The bidirectional nature of descending control is not new. What has changed is the level of resolution at which this system can now be studied. With this greater resolution, it has become increasingly possible to define how descending modulatory circuits are recruited differently across physiological and pathological conditions. Advances in anatomical tracing, cellular and molecular profiling, circuit manipulation, and human neuroimaging have refined existing models of descending modulation, revealing greater circuit diversity, neurochemical specificity, and state dependence than classical frameworks could fully capture. These developments make it timely to revisit descending pain modulation through a contemporary lens, integrating canonical brainstem pathways with newly identified circuits, local network interactions, and translational approaches across acute, inflammatory, neuropathic, and nociplastic pain states.

## 2. Why Revisit Descending Pain Modulation Now?

### 2.1. From Established Principles to Current Mechanistic Questions

Descending pain modulation has long been recognized as a central organizing principle of pain neurobiology [1,2]. Experimental and clinical work established that nociceptive transmission is not passively relayed from the periphery to the brain, but is continuously shaped by descending influences that can suppress or amplify spinal nociceptive processing [3,4,5]. Classical work on the periaqueductal gray (PAG), rostral ventromedial medulla (RVM), locus coeruleus (LC), dorsal reticular nucleus (DRt), and related brainstem structures made it clear that descending control cannot be reduced to a uniformly analgesic mechanism. Instead, it reflects a dynamic balance between inhibitory and facilitatory processes [1,4,5,6,7,8].

The reason to revisit this field is to ask how that control is implemented across defined circuits, cell populations, neurochemical systems, and functional states [2,9,10]. Questions once framed in broad physiological terms can now be asked more precisely. Which descending pathways are engaged across different pain states? How do distinct neuronal populations within the same structure generate opposing modulatory outputs? How do local brainstem interactions shape or constrain descending control? Which changes reflect adaptive flexibility, and which instead mark maladaptive reorganization associated with pain chronification?

These questions have become increasingly important because chronic pain is now understood less as a simple consequence of persistent peripheral input and more as a disorder of distributed circuit function involving altered sensory, affective, cognitive, and modulatory processing [2,9,11,12]. Traditional conceptual oppositions, such as inhibition versus facilitation or top-down control versus spinal processing, are no longer sufficient to capture the complexity of current evidence [2,9,11]. The contemporary challenge is to define how distinct circuit elements are organized, recruited, and reweighted across physiological and pathological conditions.

### 2.2. Recent Advances in Circuit, Cellular, and Translational Research

Recent methodological advances have changed how descending pain modulation can be investigated. Strategies such as optogenetic and chemogenetic manipulations, molecular profiling, cell-type-specific targeting, and large-scale connectomic approaches have enabled finer dissection of descending circuits than was possible with classical lesion, stimulation, or pharmacological methods alone [2,9,10,13]. These approaches have refined our understanding of long-studied systems such as the PAG–RVM axis, descending serotonergic pathways, and LC–spinal noradrenergic projections. They have also drawn attention to additional descending routes and local network interactions that contribute to pain modulation [2,9,11].

This contemporary literature has several important implications. First, anatomical regions previously treated as functionally unified are now understood to contain heterogeneous neuronal populations with distinct and sometimes opposing effects on nociceptive processing [6,9,12,14]. Second, descending output appears strongly state-dependent, varying with stimulus context, arousal, stress, inflammation, injury, behavioral demands, and prior experience [2,9,12,15,16]. Third, local microcircuits and reciprocal interregional interactions have become central to understanding modulatory function, challenging simplified hierarchical models in which pain control is viewed as a purely top-down process [2,9,15].

At the same time, advances in human pain research have expanded the translational relevance of descending pain modulation [2,13,17]. Experimental paradigms such as conditioned pain modulation (CPM), a human psychophysical measure of how one noxious stimulus modulates the perceived intensity of another, and temporal summation, together with pharmacological probing of monoaminergic and opioidergic systems and advanced neuroimaging approaches, have provided convergent, albeit indirect, evidence that descending modulatory processes are altered across a wide range of pain conditions [11,18,19].

Human studies now provide a stronger basis for linking mechanistic models derived from experimental neuroscience to clinically meaningful variation in pain sensitivity, symptom profiles, and treatment response [13,18,19,20]. Even so, major interpretative challenges remain. Human measures generally capture integrated system-level outputs rather than the activity of discrete pathways, and the relationship between circuit-specific findings in animal models and multidimensional human phenotypes is often complex [2,11,17,19]. For this reason, any updated account of descending pain modulation must be both mechanistically informed and translationally careful.

### 2.3. Scope and Objectives of This Review

The aim of this review is to synthesize current understanding of descending pain modulation, with particular attention to circuit mechanisms and translational evidence [2,9,13]. Rather than revisiting the field primarily as a historical progression from inhibitory models toward bidirectional control, we focus on the contemporary questions that now define it: circuit specificity, cellular diversity, neurochemical heterogeneity, state dependence, network integration, and translational interpretation across pain states [2,9,11]. We examine canonical pathways, including the PAG–RVM system and LC–spinal noradrenergic projections, together with the established dorsal reticular nucleus (DRt) system and more recently identified descending circuits that broaden current views of modulatory control [1,7,9,10,21].

A second objective is to bridge experimental neurobiology and human pain research [2,17,18]. To this end, we discuss how insights from circuit and cellular studies can inform the interpretation of CPM, temporal summation, pharmacological modulation, and human neuroimaging, while emphasizing the conceptual limits of one-to-one translation between experimental pathways and human measures [9,11,18,19,22]. This is especially relevant in chronic pain, where impaired inhibitory flexibility, enhanced facilitatory bias, and altered context dependence may coexist in ways not captured by simple loss-of-inhibition or gain-of-facilitation accounts.

Finally, this review seeks to provide a framework that is mechanistically grounded and clinically relevant. By viewing descending pain modulation as a distributed and dynamically reconfigurable system, we aim to clarify how its reorganization may contribute to pain chronification, inter-individual variability, and heterogeneity of treatment response [2,11,12]. In this sense, revisiting descending modulation is not merely an exercise in updating established knowledge. It is a necessary step toward more precise pain phenotyping and more mechanism-informed therapeutic strategies.

### 2.4. Narrative Review Approach

This article was designed as a narrative, mechanism-oriented review rather than as a systematic or scoping review. Its purpose was not to provide an exhaustive catalogue of all studies on descending pain modulation, but to critically synthesize evidence relevant to the organization, state dependence, neurochemical plasticity, and translational interpretation of descending modulatory systems across pain states.

The literature was identified through targeted searches of PubMed/MEDLINE, Web of Science, and Google Scholar, complemented by citation tracking from key primary studies and major reviews. Searches combined terms related to descending pain modulation, descending inhibition, descending facilitation, periaqueductal gray, rostral ventromedial medulla, locus coeruleus, dorsal reticular nucleus/subnucleus reticularis dorsalis, conditioned pain modulation, diffuse noxious inhibitory controls, temporal summation, chronic pain, neuropathic pain, inflammatory pain, nociplastic pain, neuroimaging, and pain phenotyping. Foundational studies were included when historically or mechanistically important, whereas recent literature was prioritized when it provided updated circuit-level, neurochemical, imaging, or translational evidence.

Because the review is narrative and interpretative, study selection was guided by mechanistic relevance and conceptual contribution rather than by formal systematic-review criteria. The figures and integrative models presented below should therefore be understood as author-derived conceptual syntheses of the literature rather than empirically validated pathway maps. All figures were generated with the assistance of SciFig (https://scifig.ai; web version, accessed July 2026; no version number is assigned to this continuously updated service) and ChatGPT (GPT-5, OpenAI) and were subsequently reviewed, edited, and refined by the authors through successive rounds of revision to ensure scientific accuracy, conceptual coherence, and consistency with the literature.

## 3. Conceptual Framework of Descending Pain Modulation

### 3.1. From Gate Control to Bidirectional Modulation

Descending pain modulation is best understood as a core organizing principle of nociceptive processing, rather than as a corrective mechanism engaged only under special circumstances [1,2,23]. Classical models of pain established that nociceptive transmission is not a passive relay from peripheral receptors to the brain, but is subject to central regulation at multiple levels [3,4]. Early formulations such as gate control theory provided the conceptual basis for this view by framing spinal nociceptive processing as dynamically modifiable rather than fixed [3].

Subsequent work on brainstem pain-modulating circuits, particularly those centered on the periaqueductal gray (PAG) and rostral ventromedial medulla (RVM), extended this principle and showed that descending systems can profoundly alter spinal nociceptive gain [5,9]. Over time, it became clear that these influences are not exclusively inhibitory. Instead, descending pathways can suppress or facilitate nociceptive transmission depending on behavioral context, physiological state, and circuit recruitment [1,5,7,24]. Descending modulation is therefore no longer treated as synonymous with endogenous analgesia, but as a bidirectional regulatory function embedded within broader systems for survival, salience, and behavioral control [2,8,9].

This historical shift remains important, but it is no longer the main question. The central question is how such opposing inhibitory and facilitatory outputs are generated, coordinated, and reconfigured across pain states [5,9]. In this sense, the conceptual transition from “descending analgesia” to “descending modulation” should be seen not as the endpoint of the field, but as the foundation for more precise mechanistic questions.

### 3.2. Inhibition and Facilitation as Interacting Processes

Descending inhibition and descending facilitation are better regarded as interacting processes that are continuously available to the organism, rather than as mutually exclusive states [1,5,9]. In this view, pain modulation reflects the net output of multiple systems whose relative contributions vary across circumstances [9,11]. Inhibition and facilitation should therefore not be treated as simple opposites located at the ends of a linear continuum. They are better understood as partially overlapping and dynamically coordinated modes of control [2,5,11].

This distinction matters because persistent pain rarely reflects a pure gain of facilitation or a pure loss of inhibition in isolation. More often, chronic pain states appear to involve altered weighting of interacting modulatory influences, with reduced inhibitory flexibility, enhanced facilitatory recruitment, or both [9,11,12]. This perspective also avoids reducing chronic pain to the failure of a single analgesic pathway, emphasizing instead the altered balance among several descending systems.

The RVM remains one of the clearest physiological demonstrations of this principle, with OFF-cells suppressing and ON-cells facilitating nociceptive processing [5,6,9]. Yet even in this canonical system, the significance of inhibition and facilitation lies not only in the existence of opposing cell classes. It also lies in how these populations integrate descending and local inputs under changing behavioral and sensory conditions [2,9]. In this sense, descending modulation is better understood as flexible control of nociceptive gain than as the toggling of a simple inhibitory switch.

### 3.3. State Dependence, Context, and Behavioral Relevance

A central implication of current work is that descending pain modulation is not fixed, but inherently state-dependent [2,9,11]. The same anatomical region or transmitter system may contribute to distinct, even opposing, outcomes depending on the organism’s physiological condition, motivational priorities, recent emotionally salient experiences, and broader neuroimmune state [2,9,11,25]. This principle has long been recognized in relation to stress-induced analgesia, defensive behavior, and opioid control of pain, but recent circuit-based studies have made state dependence a more explicit mechanistic theme [9,16,25].

Rather than asking whether a given descending pathway is generally inhibitory or facilitatory, it is now more informative to ask under which conditions that pathway is recruited, which neuronal populations are engaged, and what behavioral function the resulting modulation serves. This becomes especially important when considering the transition from acute to persistent pain [2,9,11,12].

In acute settings, descending control may support adaptive flexibility by transiently suppressing pain when escape, defense, or goal-directed behavior takes precedence, or by enhancing nociceptive salience when tissue protection and learning are more important [16,25,26]. In more persistent pain states, however, the same systems may be reconfigured in ways that favor ongoing facilitatory bias, reduced inhibitory reserve, or impaired contextual adaptability [9,11,12,27]. State dependence thus links the neurobiology of descending modulation to broader questions of pain chronification and helps explain why similar circuitry can participate in adaptive modulation in one setting and maladaptive amplification in another.

Behavioral relevance is equally central [2,26]. Pain is not regulated in isolation, but in relation to competing survival demands, attentional priorities, affective significance, and action selection [13,17,26]. Descending systems are thus embedded within a broader control architecture that calibrates nociceptive processing according to what matters most to the organism at a given moment. This places descending modulation within the logic of behavioral regulation rather than within a narrow analgesic framework.

### 3.4. Descending Modulation as Distributed Network Function

A major conceptual refinement has been the move away from viewing descending modulation as the output of a single hierarchical pathway. Although the PAG–RVM axis remains central, current models increasingly describe descending control as a distributed network function spanning forebrain, brainstem, and spinal circuits [11,13,17]. Within this network, the RVM acts as a major output node. Its activity is shaped by convergent top-down and bottom-up influences, including direct nociceptive input from the dorsal horn, indirect input relayed through structures such as the parabrachial complex, local circuit interactions, and broader interregional dynamics [5,9,28]. This distributed view better accommodates the anatomical diversity and functional complexity now recognized in descending modulation, including contributions from noradrenergic, serotonergic, opioidergic, and other systems that cannot be fully captured by a single-pathway model [1,2,9,11].

This network perspective also helps clarify an important interpretative issue. Terms such as “top-down modulation” remain useful, but they can be misleading if taken to imply that pain control is imposed unidirectionally from higher centers onto passive lower relays. In reality, descending modulation emerges from reciprocal and context-sensitive interactions across multiple levels of the neuraxis [2,9,13,17]. Conceptually, descending pain modulation is therefore better understood as an emergent systems function than as the action of an isolated analgesic center.

Taken together, these considerations suggest that descending pain modulation is best viewed as a distributed, state-dependent, and behaviorally embedded process arising from interacting inhibitory and facilitatory systems. The classical top-down model remains useful but is no longer sufficient. Descending modulation is not a command pathway imposed by higher centers onto passive lower relays, but a distributed control system in which spinal, brainstem, and forebrain processes continuously reshape one another. This view preserves the strengths of classical models while moving beyond their linear architecture, and provides the conceptual basis for the more detailed circuit, network, and pain-state analyses developed in the sections that follow.

## 4. Circuit Organization of Descending Pain Modulation

**Figure 1:**
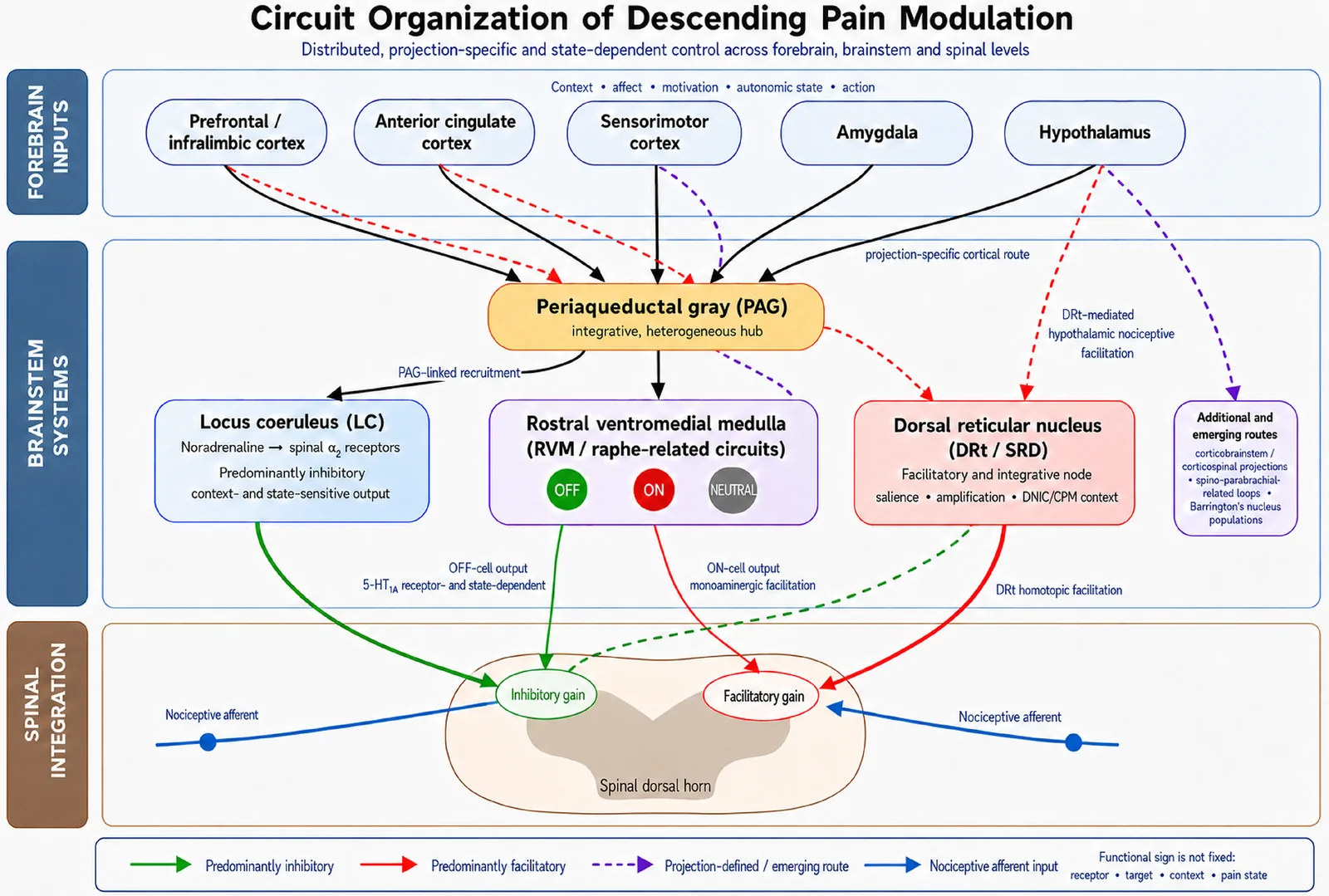
Circuit organization of descending pain modulation. Descending pain control is represented as a distributed, projection-specific, and state-dependent network spanning forebrain, brainstem, and spinal levels. Prefrontal/infralimbic, anterior cingulate, sensorimotor, amygdalar, and hypothalamic inputs convey contextual, affective, motivational, autonomic, and action-related information to brainstem modulatory systems. The periaqueductal gray (PAG) acts as a heterogeneous integrative hub that recruits the rostral ventromedial medulla (RVM), locus coeruleus (LC), dorsal reticular nucleus (DRt/SRD), and additional projection-defined descending routes. Within the RVM, OFF-, ON-, and NEUTRAL-cell populations illustrate the coexistence of inhibitory, facilitatory, and context-dependent microcircuit outputs. LC–spinal noradrenergic projections are shown as predominantly inhibitory through spinal α_2_-adrenoceptor mechanisms, whereas RVM ON-cell and DRt/SRD outputs contribute to facilitatory gain. The DRt/SRD is represented as a facilitatory and integrative node involved in salience-dependent amplification, homotopic facilitation, and DNIC/CPM-related network configurations. At the spinal dorsal horn, convergent descending influences dynamically regulate inhibitory and facilitatory gain imposed on nociceptive afferent input. Green arrows indicate predominantly inhibitory influences, red arrows predominantly facilitatory influences, purple dashed arrows projection-defined or emerging routes, blue arrows nociceptive afferent input, and black arrows structural or integrative projections. The functional sign of a pathway is not fixed, but depends on receptor subtype, projection target, behavioral context, and pain state. CPM, conditioned pain modulation; DNIC, diffuse noxious inhibitory controls. This is an author-derived conceptual schematic and should not be interpreted as an empirically validated pathway map. The figure was generated with the assistance of SciFig.AI (https://scifig.ai) and ChatGPT and was subsequently reviewed, edited, and refined by the authors through successive rounds of revision to ensure scientific accuracy, conceptual coherence, and consistency with the literature.

The principal components of descending pain modulation are organized within a distributed, projection-specific, and state-dependent network that integrates forebrain, brainstem, and spinal processes, as schematically summarized in Figure 1.

### 4.1. Periaqueductal Gray as an Integrative Hub

The PAG remains a central organizing node within descending pain modulation because of its strategic position at the interface of forebrain appraisal systems and brainstem output pathways [5,9,29]. It receives convergent input from cortical, limbic, hypothalamic, and brainstem structures and, in turn, regulates multiple downstream modulatory systems, most notably the rostral ventromedial medulla (RVM), as well as noradrenergic nuclei and other brainstem structures [5,29]. This anatomical arrangement allows the PAG to translate contextual, affective, autonomic, and motivational information into changes in nociceptive gain [5,17,29].

Although classically associated with analgesia, the PAG is no longer viewed as a unitary antinociceptive structure [4]. Contemporary work instead supports a functionally heterogeneous organization in which different PAG subregions, projection targets, and neuronal populations contribute to distinct modulatory outcomes depending on behavioral state and circuit recruitment [5,9,14,30].

This distinction matters conceptually. The PAG should not be treated simply as the first relay of a canonical descending analgesia pathway, but as an integrative structure through which multiple internal and external signals are routed into descending control [5,9,17,30]. Recent studies support this view by showing that cortical projections can engage PAG-linked pathways with considerable specificity [10]. In mice, distinct primary sensorimotor cortical subpopulations project to the PAG and RVM, with PAG-linked pathways recruiting downstream antinociceptive systems in ways that depend on projection target and behavioral state [10]. These observations reinforce the view that PAG function depends on projection-defined organization and behavioral state.

Human evidence, although necessarily less mechanistically precise, is beginning to align with this more differentiated view. Recent ultrahigh-field imaging data indicate that placebo-related analgesic responses can engage somatotopically organized circuitry involving the lateral PAG and the RVM, suggesting that functionally meaningful internal organization can now be detected in human brainstem pain circuitry [22]. This spatially differentiated view is further supported by evidence that PAG signal changes during CPM are associated with both hypoalgesic and hyperalgesic responses, and that distinct PAG columnar territories may contribute differentially to these outcomes, with lateral/dorsolateral PAG linked to hypoalgesia and ventrolateral/lateral PAG to hyperalgesia [31]. This has important translational implications because it supports the idea that descending control in humans may recruit classical brainstem systems in a spatially structured and body-site-specific manner.

### 4.2. Rostral Ventromedial Medulla Microcircuits: ON-, OFF-, and NEUTRAL-Cell Populations

Among brainstem structures involved in descending pain modulation, the RVM remains one of the clearest examples of bidirectional control implemented through functionally defined output populations [5,6,9]. It integrates top-down and bottom-up influences that shape the activity of OFF-cells and ON-cells, which respectively suppress and facilitate nociceptive processing [5,9]. This represents one of the most robust physiological frameworks in pain neurobiology and continues to anchor mechanistic interpretations of descending modulation.

At the same time, recent work has made clear that the classical ON/OFF framework is not the endpoint of the story. The field now faces the challenge of relating these functionally defined populations to genetically and molecularly identified cell classes, and progress has been less straightforward than initially hoped [9]. Contemporary reviews emphasize that attempts to assign single molecular markers to RVM pain-modulating populations have produced partly conflicting behavioral findings. Although ON- and OFF-cells remain robust physiological categories, their molecular definition remains unresolved, reflecting the absence of simple cell markers for these populations and the complex distribution of receptors and transmitters across RVM neurons [9,32]. Recent circuit-based work further suggests that molecularly defined RVM ensembles do not map directly onto the classical ON/OFF scheme and may contribute to antinociception through parallel cell populations and circuit configurations that are richer than a simple binary model implies [33]. This underscores that molecular identity and physiological function do not map onto each other in a simple one-to-one manner. Human ultrahigh-field imaging now supports this more nuanced interpretation by showing that RVM signal changes are associated with both hypoalgesic and hyperalgesic CPM responses, and may correlate with the magnitude of pain change rather than simply distinguishing one response class from the other [31]. This is not a weakness of the ON/OFF framework, but rather a sign that RVM organization is richer than a purely binary scheme.

In this context, NEUTRAL cells deserve brief consideration. Although less prominently discussed than ON- and OFF-cells, they remain relevant because they indicate that not all RVM neurons are directly linked to reflex-associated bidirectional control [5,6,34,35]. Their existence supports the view that the RVM is not merely a two-channel output device, but a dynamic microcircuit in which pain-modulating neurons coexist with populations whose roles may be permissive, state-dependent, or only indirectly related to nociceptive output [5,9,35]. The most balanced position is to retain ON- and OFF-cells as the dominant functional framework for descending modulation, while acknowledging that NEUTRAL populations and broader population dynamics likely contribute to network regulation, contextual recruitment, and the shaping of final descending output.

A further point worth emphasizing is that the ON/OFF logic should not be generalized too loosely to all descending structures [5,9]. The RVM remains indispensable not because it provides a simple ON/OFF template for the whole field, but because it shows how bidirectional modulation can be implemented by organized brainstem microcircuits.

### 4.3. Descending Serotonergic Pathways: Receptor-Specific and State-Dependent Actions

Serotonergic descending modulation remains one of the most complex components of the field because serotonin cannot be assigned a uniform inhibitory or facilitatory role [1,9,19,36]. Serotonergic influence, much of which arises from medullary raphe-related circuits that overlap functionally with the RVM system, contributes to both descending facilitation and inhibition through multiple spinal receptor subtypes with divergent effects on nociceptive transmission [1,19,37,38,39]. Current work therefore increasingly emphasizes receptor-specific and state-specific interpretations, rather than global statements about serotonin as analgesic or pronociceptive.

This complexity is especially important in persistent pain. Serotonin can participate in inhibition under some circumstances, yet contribute to facilitation or maintenance of hypersensitivity in others, depending on receptor distribution, circuit context, and pathophysiological state [11,19,37,40]. This is why serotonergic descending control is such a useful example of the broader conceptual shift in pain modulation. What matters is not the transmitter alone, but which cells release it, where it acts, which receptors are engaged, and under which state conditions the system is recruited.

Accordingly, the serotonergic component of descending modulation is better understood as a receptor-diverse modulatory ensemble embedded within larger brainstem circuits [9,19]. In this respect, serotonergic signaling illustrates why descending pathways cannot be interpreted at the transmitter level alone.

### 4.4. Locus Coeruleus–Spinal Noradrenergic Pathways: Inhibition, Facilitation, and Mechanism

The LC–spinal noradrenergic system remains a major descending pathway through which nociceptive processing can be modulated, particularly in relation to inhibition [41,42,43]. Its classical antinociceptive role is strongly linked to spinal noradrenaline acting at α2-adrenoceptors, where it reduces nociceptive transmission through presynaptic and postsynaptic mechanisms [41,42,44,45]. This basic framework is still valid and continues to underpin the therapeutic rationale for drugs that enhance noradrenergic tone in chronic pain, particularly through mechanisms involving spinal α2-adrenoceptors. However, the efficacy of such approaches depends on the functional state of the broader descending modulatory system [23,42,43,46]. Current conceptual models also emphasize that LC function cannot be reduced to a single inhibitory output, since noradrenergic effects are shaped by receptor subtype, spinal target, circuit state, and chronic pain-related network reorganization [9,42,43,46,47].

This distinction is central to clarifying the dual role of the LC. Under many conditions, LC recruitment contributes to descending inhibition and analgesia, particularly during stress- or behavior-linked modulation [41,42]. However, the same system can become less protective, insufficient, or even functionally permissive of facilitation when the underlying network state changes [9,11,43,48,49]. This interpretation is consistent with recent 7T fMRI evidence showing that the locus coeruleus is engaged during both CPM hypoalgesia and hyperalgesia, reinforcing the view that its contribution is not fixed in sign but depends on the broader network configuration and response phenotype [31]. The point is not simply that the LC “switches sides.” Rather, noradrenergic modulation is filtered through receptor-specific effects, altered recruitment patterns, and chronic pain-related reorganization of distributed descending circuits. In addition, LC contributions to pain appear increasingly intertwined with arousal, stress responsivity, and pain-associated affective comorbidity, reinforcing the need to interpret LC-dependent modulation in broader behavioral and network terms rather than as a fixed antinociceptive channel. Viewed this way, the LC is best understood as a context-sensitive modulatory system whose output depends on the interaction between intrinsic circuit organization and ongoing physiological or pathological state [9,42,49].

Recent studies reinforce this network perspective by showing that PAG- and RVM-linked pathways can be embedded within long-range descending architectures through which cortical control engages brainstem antinociceptive systems [10,17]. Within this perspective, the LC is best positioned as part of a distributed descending system in which cortex, PAG, RVM, LC, and spinal mechanisms interact to shape final nociceptive output.

### 4.5. Dorsal Reticular Nucleus as a Facilitatory and Integrative Node

The dorsal reticular nucleus (DRt), also historically referred to as the subnucleus reticularis dorsalis (SRD), provides a strong mechanistic counterpoint to the longstanding dominance of PAG-centered inhibitory models [7,8]. The DRt is best understood as a facilitatory and integrative node within descending pain modulation, capable of enhancing nociceptive transmission and amplifying behaviorally relevant input [7,50]. Its significance lies not only in its descending projections to the spinal dorsal horn, but also in its position within reciprocal networks linking spinal, cortical, limbic, hypothalamic, and brainstem structures [8,50,51,52,53,54].

The emphasis placed on the DRt/SRD in this review reflects its distinctive position as one of the best-characterized medullary systems linking nociceptive convergence, descending facilitation, and heterotopic modulatory control. Early independent studies by Villanueva et al. provided foundational evidence regarding the convergence and encoding properties of SRD neurons and their role in pain transmission and modulation [55,56,57]. Subsequent anatomical, functional, and neurochemical studies, discussed below, further defined its reciprocal spinal organization, facilitatory output, supramedullary recruitment, and chronic pain-related plasticity.

Early electrophysiological studies showed that the DRt contains neurons that respond selectively to noxious stimuli from widespread areas of the body and exhibit convergence of nociceptive inputs from the entire body surface [55,56]. These neurons also encode the intensity of electrical, thermal, and mechanical noxious stimulation, indicating that the DRt contributes actively to the processing of convergent nociceptive information rather than functioning as a simple relay [56,57]. In parallel, anatomical studies indicate that lamina I spinal neurons project to the DRt and that DRt neurons projecting to lamina I participate in a reciprocal spino–bulbo–spinal organization, further supporting the view that the DRt functions as an integrative and facilitatory node within descending pain modulation [7,8,50,58,59].

Experimental work supports the view that the DRt contributes actively to descending facilitation rather than simply reflecting reduced inhibitory control elsewhere. Pharmacological activation of the DRt enhances spinal nociceptive responsiveness, whereas lesions or functional disruption attenuate hyperalgesic behavior in acute and inflammatory pain paradigms [7,60,61]. In the DRt, descending facilitation is not inferred from the absence of inhibition; it can be experimentally produced, interrupted, and mechanistically traced. Functionally, the DRt is well positioned to increase the gain of salient or behaviorally relevant nociceptive input, reinforcing protective responses when rapid selection of incoming signals is advantageous.

The DRt also appears capable of sustaining amplified nociceptive output through dynamic local and network mechanisms. Glutamatergic activation of the DRt enhances the responses of deep dorsal horn wide-dynamic range (WDR) neurons, particularly by increasing post-discharge and strengthening wind-up-related excitability, supporting the view that this nucleus can amplify spinal nociceptive signaling once engaged [62]. Related work in the DRt has demonstrated wind-up properties and suggested that such amplification is not merely a passive reflection of spinal wind-up, but may be locally generated and shaped by intrinsic excitability and tonic inhibitory control within the nucleus itself [63]. At the circuit level, more recent genetic and chemogenetic studies have identified excitatory spinal–brainstem–spinal configurations involving the parabrachial nucleus and medullary dorsal reticular formation that transform transient nociceptive input into sustained nocifensive behavior, reinforcing the idea that DRt-related facilitation should be understood within distributed excitatory networks rather than as the output of an isolated relay [64]. Together, these data strengthen the view that the DRt is part of a dynamically recruitable network capable of prolonging and amplifying nociceptive signaling.

A further reason to emphasize the DRt is that it provides a clear route through which supramedullary structures can recruit descending facilitation. Experimental studies indicate that forebrain and hypothalamic influences can be relayed through the DRt to enhance spinal nociceptive processing. Activation of the anterior cingulate cortex has been shown to facilitate spinal nociception through a DRt-dependent mechanism, and lesion of the DRt blocks ACC-induced facilitation [65]. Likewise, galanin-mediated hyperalgesia from the dorsomedial hypothalamus engages a descending facilitatory pathway relaying in the DRt, whereas the pronociceptive effect of infralimbic cortex mGluR5 activation is blocked by lidocaine or GABAergic inhibition in the DRt [66,67]. These findings are especially important because they show that descending facilitation is not a vague residual phenomenon but can be recruited by anatomically defined cortical and hypothalamic circuits. The DRt therefore functions as an interface through which motivational, affective, contextual, and sensorimotor regulation can bias pain processing toward amplification.

**Figure 2:**
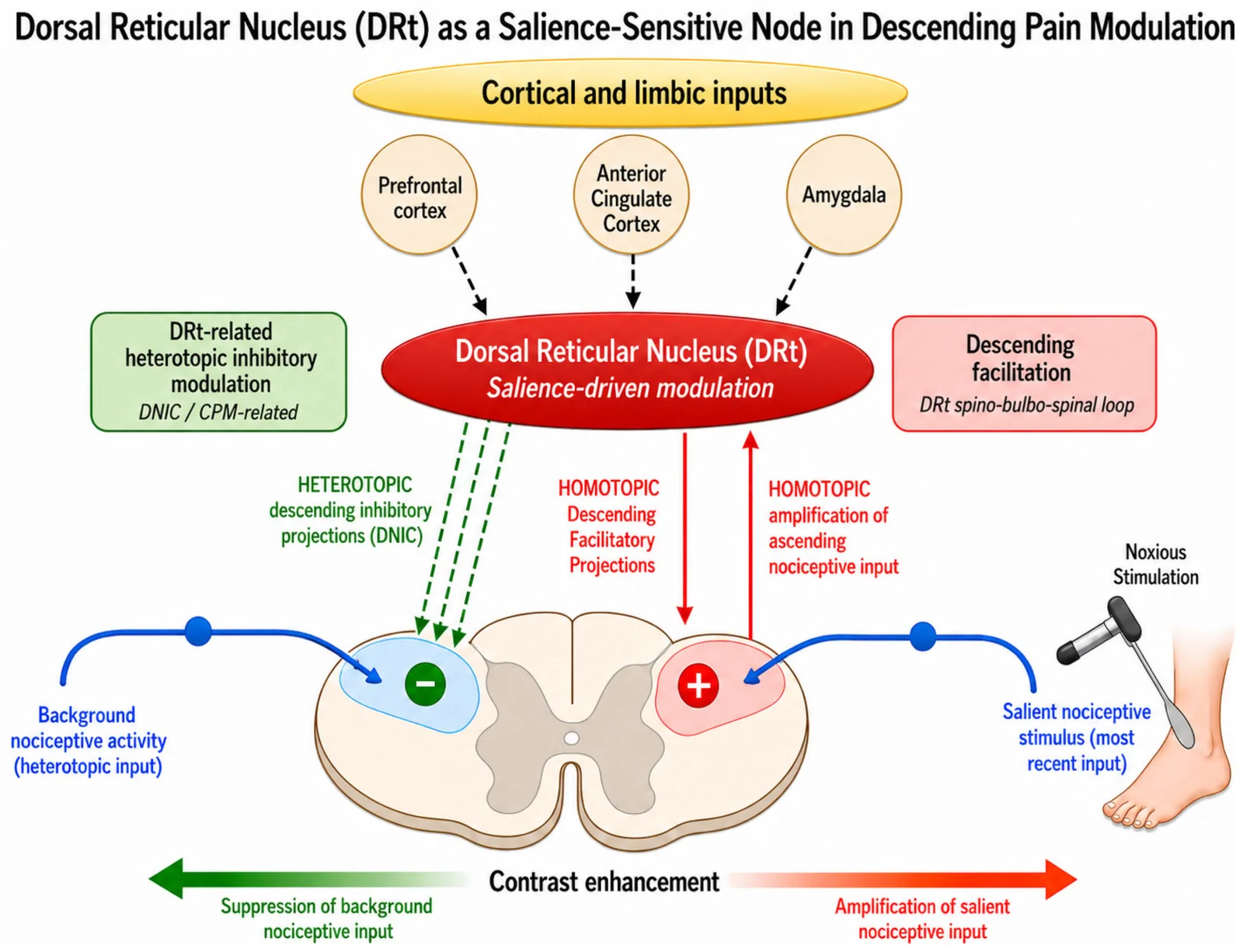
Dorsal reticular nucleus as a salience-sensitive node in descending pain modulation. The dorsal reticular nucleus (DRt) integrates cortical and limbic influences from the prefrontal cortex, anterior cingulate cortex, and amygdala with ascending nociceptive information. The scheme illustrates a proposed contrast-enhancement function in which DRt recruitment preferentially amplifies recent, homotopic, and behaviorally salient nociceptive input through reciprocal spino–bulbo–spinal circuitry and descending facilitatory projections to the ipsilateral spinal dorsal horn. In parallel, DRt-related network configurations may contribute to DNIC/CPM-associated suppression of ongoing, spatially remote heterotopic nociceptive activity. These facilitatory and inhibitory effects should not be interpreted as fixed or mutually exclusive outputs of an isolated nucleus, but as context-dependent expressions of distributed modulatory networks involved in stimulus selection and nociceptive signal prioritization. Blue pathways represent primary nociceptive afferent input, red pathways represent homotopic facilitatory modulation and amplification, and green pathways represent heterotopic inhibitory modulation. The red plus (+) and green minus (−) symbols indicate, respectively, facilitation/amplification and inhibition/suppression of nociceptive processing in spinal dorsal horn neurons. The resulting functional effect is enhanced contrast between newly salient nociceptive input and competing background nociceptive activity. CPM, conditioned pain modulation; DNIC, diffuse noxious inhibitory controls. This is an author-derived conceptual schematic and should not be interpreted as an empirically validated pathway map. The figure was generated with the assistance of SciFig.AI (https://scifig.ai) and ChatGPT and was subsequently reviewed, edited, and refined by the authors through successive rounds of revision to ensure scientific accuracy, conceptual coherence, and consistency with the literature.

Importantly, the facilitatory role of the DRt should not be interpreted in rigidly linear terms. A more nuanced account is required because the DRt has also been implicated in diffuse noxious inhibitory controls (DNIC), whereby one noxious stimulus suppresses responses to another, spatially remote noxious input. Lesion experiments showed that disruption of the DRt significantly reduces the magnitude of DNIC, indicating that this nucleus is one of the supraspinal relays involved in the circuitry underlying heterotopic inhibitory control [68]. This does not negate the predominantly facilitatory effects observed during direct activation or disinhibition [7,8]. Rather, it suggests that the DRt participates in network configurations whose functional meaning depends on context. The established findings therefore support DRt/SRD involvement in both descending facilitation and heterotopic inhibitory modulation, whereas the proposal that these functions may converge to support contrast enhancement and stimulus prioritization should be understood as an integrative interpretation advanced here. One plausible interpretation is that DRt recruitment may reinforce recent, homeotopic, or behaviorally salient nociceptive input. Under appropriate DNIC-like conditions, the same circuitry may also contribute to the suppression of ongoing, previously induced, heterotopic, or competing background nociceptive activity. In this way, the DRt may enhance the contrast between newly salient nociceptive input and ongoing background activity, thereby helping to focus the behavioral response on the most relevant stimulus. In this view, facilitation and participation in inhibitory control are not mutually exclusive. Instead, they may reflect a shared role in stimulus selection, signal prioritization, and optimization of nociceptive signal-to-noise relationships across behavioral contexts. Recent ultrahigh-field human imaging further supports the relevance of the DRt to CPM by showing brainstem signal changes associated with both hypoalgesic and hyperalgesic response profiles, reinforcing the view that this medullary region remains a meaningful component of human pain-modulatory circuitry rather than a purely preclinical construct [31]. Accordingly, the DRt may contribute to contrast enhancement by simultaneously amplifying recent or behaviorally salient nociceptive input and suppressing competing heterotopic background activity, as schematically represented in Figure 2.

Finally, the DRt provides a particularly useful bridge from acute and inflammatory modulation to persistent pain and circuit reorganization. In neuropathic conditions, blockade of the DRt reduces several expressions of spinal sensitization, including spontaneous hyperactivity, responses to heterotopic stimulation, and particularly the afterdischarges of WDR neurons, supporting the view that DRt-dependent facilitation contributes directly to the maintenance of central sensitization rather than merely to transient amplification of nociceptive input [69]. This interpretation is consistent with earlier evidence that glutamatergic activation of the DRt enhances dorsal horn WDR neuronal responses primarily by increasing post-discharge and strengthening wind-up-related excitability, thereby supporting a facilitatory role of this nucleus in sustained spinal nociceptive amplification [62]. This is highly relevant because it shows that chronic pain may involve not only reduced inhibitory reserve but also active facilitatory organization within defined brainstem circuits. The DRt is therefore not peripheral to a modern view of descending pain modulation. On the contrary, it is one of the clearest examples of why contemporary models must move beyond an inhibition-centered narrative and explicitly account for networked forms of descending facilitation, state dependence, and circuit reweighting across pain conditions. For this reason, an account of descending modulation that treats the DRt/SRD as peripheral risks missing one of the clearest examples of organized descending facilitation and state-dependent circuit reweighting.

### 4.6. Additional Descending Pathways and Recent Circuit-Level Refinements

Older pathway-limited models are no longer sufficient. Additional descending routes and cortical control systems are now recognized as important contributors to nociceptive modulation [2,9,23,70,71]. Beyond the canonical PAG–RVM and LC-centered systems, attention has expanded to corticobrainstem and corticospinal contributions, ACC-linked descending facilitatory routes, spino–parabrachial–reticular loops, and newly identified pontine descending populations with selective sensory and behavioral functions [10,21,64,72]. In parallel, recent circuit-level studies have refined previously recognized descending pathways by resolving their projection-specific, neurochemical, or state-dependent organization [16,73,74]. This does not diminish the classical pathways; it places them in a more differentiated circuit context. Recent work using activity-dependent genetic labeling, viral tracing, calcium imaging, and chemogenetic manipulation has extended this logic to cortical microcircuits, identifying an intra-somatosensory S2→S1 feedforward inhibitory circuit that mediates pain-induced analgesia in mice [75]. Although this circuit is not itself a classical descending brainstem pathway, it reinforces the point that analgesia can emerge from projection-defined and state-recruited circuits outside the traditional descending brainstem model.

A particularly relevant example is the role of primary sensorimotor cortex in descending pain modulation. Recent evidence indicates that distinct sensorimotor cortical populations project either to the PAG or directly to the RVM and can influence descending antinociceptive systems in ways linked not only to pain processing but also to motor behavior and locomotor state [10]. This circuit-level evidence converges with translational neuromodulation literature showing that the primary motor cortex is not merely a motor output region. Rather, it is a cortical node with extensive subcortical connectivity, and its stimulation can engage limbic, cortical, subcortical, and descending modulatory systems relevant to analgesia [76]. In humans, this sensorimotor framing has recently been strengthened by simultaneous corticospinal fMRI evidence showing that M1–spinal cord coupling is a major component of a pain-sensitivity signature that relates sensorimotor network organization to individual differences in pain perception [77]. Together, these observations matter for two reasons. First, they show that cortical control of nociception is anatomically more specific than many earlier top-down models implied. Second, they help link descending modulation to action systems, corticospinal coupling, cortical excitability, and behavioral state, reinforcing the idea that pain control is embedded within broader sensorimotor regulation and may be accessible to mechanism-informed neuromodulation strategies.

Another line of work has identified pain-related descending neurons mapped to Barrington’s nucleus in the dorsal pontine tegmentum, expanding the known architecture of supraspinal control over nociceptive processing [21]. This pontine contribution extends the map of supraspinal pain control beyond the best-established brainstem pathways and suggests that additional descending centers may participate in state-dependent modulation of pain and related behavioral functions. It also reinforces the principle that descending control is organized through functionally specialized cell populations rather than through anatomically defined nuclei alone.

The implication is that descending pain modulation is better viewed as an expanding family of interacting circuits than as a closed set of brainstem pathways. New circuit-level studies are likely to continue identifying additional nodes, projection-defined populations, and state-specific recruitment patterns that refine current models [10,21]. These systems should be considered, even if the strongest mechanistic detail remains concentrated in the better-established PAG–RVM, LC, and DRt frameworks. Their recognition also makes clear that descending modulation cannot be understood solely in terms of isolated pathways, but requires attention to how these circuits are organized and interact across levels of the neuraxis.

## 5. Network Integration Beyond Simple Top-Down Models

### 5.1. Brainstem Microcircuits and Reciprocal Interactions

Although the major nodes of descending pain modulation can be described individually, their function cannot be understood in isolation. Descending pain modulation is often described in hierarchical terms, but such a description is now only partially adequate [2,17]. Forebrain influences on brainstem and spinal nociceptive processing remain important, but they do not fully explain pain modulation, which also depends on local processing within the brainstem itself. Brainstem nuclei involved in pain control do not simply relay descending influences. Rather, they participate in reciprocal, state-sensitive microcircuits that integrate sensory input, internal state, autonomic signals, and long-range modulation into functionally organized output [2,5,9]. The RVM remains a major output node, but its role is best understood within a web of reciprocal interactions rather than as the terminal step in a simple linear chain.

This changes how descending control is interpreted. If the system is framed too narrowly as top-down modulation, there is a risk of implying that higher structures impose pain regulation on relatively passive lower relays [17,78]. In reality, brainstem regions such as the PAG, RVM, locus coeruleus, and related reticular structures integrate both descending and ascending influences while engaging local population dynamics that shape final spinal output [5,9]. Recent experimental work has further refined this view by showing that physiologically defined pain-modulating neurons in the RVM receive both indirect nociceptive input relayed through the parabrachial complex and direct input from the trigeminal dorsal horn, indicating that bottom-up recruitment of ON- and OFF-cells is mediated through parallel pathways with distinct temporal dynamics [28]. This organization makes descending modulation less like a command hierarchy and more like a set of interacting control loops. This perspective is particularly relevant in persistent pain, where altered recruitment of facilitatory or inhibitory microcircuits may reflect not only altered forebrain drive, but also reorganization within brainstem networks themselves.

The importance of local brainstem processing is also evident in efforts to relate physiologically defined populations to underlying molecular and circuit architecture. Work on opioid modulation within descending pain-control pathways illustrates this point, showing that functionally distinct effects can depend on the cellular and circuit identity of the recruited PAG populations [14]. Functionally important output patterns do not arise from isolated neurons acting independently, but from local ensembles and reciprocal interactions that constrain recruitment, timing, and state dependence [5,9]. This is one reason why classical cell-type frameworks remain useful but incomplete: what matters is not only which population exists, but how it is embedded within local circuit structure. It is therefore more accurate to describe descending pain control as emerging from organized brainstem microcircuits than from serial relay alone.

### 5.2. Forebrain Regulation of Descending Control

Forebrain regulation remains central to descending pain modulation, but it should be described with greater anatomical and functional specificity than is possible in broad “cognitive control” formulations [2,17,78]. Cortical and limbic regions do not simply increase or decrease pain through generic top-down influence. Instead, they recruit defined descending systems through projection-specific pathways that link appraisal, salience, motor preparation, affective relevance, and autonomic state to nociceptive modulation [10,17,78]. Recent work on primary sensorimotor cortex projections illustrates this clearly, showing that distinct cortical subpopulations can differentially target the PAG and RVM, thereby engaging descending antinociceptive systems with notable circuit specificity [10].

This more precise framing matters for two reasons. First, it situates descending control within distributed behavioral regulation rather than treating it as a purely pain-specific function. Second, it avoids the misconception that forebrain influence is necessarily abstract or anatomically diffuse. Cortical control of descending modulation can be anatomically organized, functionally selective, and linked to action-related or motivational demands [10,78]. Recent causal circuit work reinforces this point by showing that placebo analgesia depends on medial prefrontal and anterior cingulate cortical input to the ventrolateral PAG, indicating that cognitively mediated pain relief can be implemented through anatomically defined cortico-brainstem descending pathways rather than through diffuse “top-down” influence alone [79]. In this sense, descending pain control is not simply imposed from “higher centers,” but assembled through specific long-range pathways that interact with local brainstem circuitry.

A similar logic applies to affective and motivational influences. Emotional salience, threat appraisal, expectation, and defensive priorities do not modulate pain by bypassing nociceptive circuitry; they act through it, recruiting distributed forebrain–brainstem networks that bias descending output according to context [17,26,78]. Recent human work also suggests that body experience may shape this modulation, since reduced CPM efficiency appears more closely related to disembodiment than to mere visual exposure to the body itself [80]. This is one of the strongest reasons to resist reducing descending modulation to a purely analgesic or purely cognitive model. The system is better understood as one in which nociceptive control is embedded within broader circuits of adaptive behavior.

### 5.3. Connectomic and Network-Level Perspectives

A major development has been the move toward connectomic and network-level interpretations of pain modulation. These perspectives do not replace classical physiological findings, but they change the scale at which those findings are integrated [9,17,78]. Rather than treating individual nuclei as isolated functional modules, this approach asks how projection-defined populations, recurrent loops, and distributed connectivity patterns shape modulatory output. In the context of descending pain modulation, this means that structures such as the PAG, RVM, LC, and DRt are better understood as network nodes whose function depends on patterns of connectivity as much as on the properties of any single region [9,10,17,78]. A recent 7T fMRI study provides a particularly compelling example of this network view, showing that CPM hypoalgesia and hyperalgesia are associated with partially overlapping but dissociable PAG, RVM, LC, A5, and SRD networks, rather than with the recruitment of a single canonical inhibitory pathway [31].

This helps explain findings that would otherwise seem contradictory. The same region may contribute to inhibition in one context and facilitation in another, not because it lacks defined function, but because it participates in different subnetworks, recruits different projection-defined populations, or operates under altered state conditions [9,11]. The connectomic perspective therefore helps explain why anatomical labels alone are often insufficient to predict modulatory outcome. Recent high-field human imaging studies reinforce this point by showing that brainstem pain-modulatory responses can be spatially organized and network-dependent, with distinct PAG–RVM or PAG–RVM–LC–A5–SRD configurations associated with different analgesic or hyperalgesic profiles [22,31].

At the translational level, a network perspective offers a useful bridge between animal circuit studies and human imaging findings [17,22]. Human neuroimaging rarely resolves the full cellular or projection-specific organization seen in experimental work. Yet it increasingly reveals coordinated brainstem and forebrain activity patterns compatible with distributed pain-modulatory systems [22,81]. From this perspective, human neuroimaging studies provide complementary information about large-scale integration and state-dependent recruitment. This is especially relevant now that human imaging is beginning to resolve spatially organized recruitment of brainstem pain-modulation regions [22,31].

### 5.4. From Hierarchical Modulation to Distributed Circuit Regulation

Taken together, these considerations refine how descending pain modulation should be understood. Descending control remains a valid term, but it should not imply a purely unidirectional or exclusively top-down process. A more accurate formulation is that nociceptive regulation emerges from distributed circuit interactions spanning forebrain, brainstem, and spinal levels, with reciprocal influences and local processing at multiple stages.

This revised framework preserves the importance of canonical pathways such as PAG–RVM and LC–spinal projections, while situating them within a broader regulatory architecture that includes brainstem microcircuits, projection-defined cortical control, and state-dependent network reconfiguration [2,9,10,21]. In that sense, moving beyond simple top-down models is not a rejection of the classical framework, but a more precise account of how descending modulation is organized and reconfigured across pain states.

This network-based perspective provides the conceptual bridge to the next section, where neurochemical systems are considered not as isolated mediators, but as modulatory substrates embedded within distributed and dynamically interacting circuits. What changes here is the unit of explanation: not a single pathway, not an isolated nucleus, and not a transmitter in itself, but the state-dependent circuit configuration in which they operate.

## 6. Neurochemical Systems Shaping Descending Modulation

### 6.1. Opioidergic Mechanisms

Opioidergic signaling remains one of the foundational neurochemical substrates of descending pain modulation [4,15,25]. Classical work established the periaqueductal gray and rostral ventromedial medulla as key loci through which endogenous and exogenous opioids can engage powerful antinociceptive control [4,5,25]. More recent work has refined this view by showing that opioid effects are exerted at multiple nodes within brainstem pain circuits rather than at a single “analgesic center” [9,15]. Opioids shape descending output through circuit-specific actions on local inhibitory interneurons, projection neurons, and functionally distinct RVM populations, thereby influencing the balance between facilitatory and inhibitory control [9,14,15,82].

This broader view matters because it prevents overly simple equations between opioids and analgesia. Opioidergic mechanisms are undeniably central to endogenous pain control, but their behavioral effects depend on where in the circuit they act, which neuronal populations are recruited, and under which physiological or pathological conditions the system is engaged [5,15,25]. In this sense, opioidergic modulation exemplifies a more general principle in descending pain control: transmitters do not determine outcome in isolation; their effects depend on the state-dependent circuitry in which they act. This becomes especially important in chronic pain, where opioidergic regulation may be reorganized in ways that alter inhibitory reserve, facilitatory drive, or the response to analgesic drugs [11,15,83].

Opioidergic modulation can no longer be discussed only in relation to PAG–RVM analgesia. Evidence from the DRt indicates that opioids also act within facilitatory descending circuits and may shape the balance between adaptive inhibition and maladaptive amplification in highly state-dependent ways [15]. In this respect, the DRt provides an especially informative example because it links opioidergic control to a brainstem region with a predominantly facilitatory profile. In neuropathic pain, increased enkephalin levels, reduced MOR-labeled cells, and increased phosphorylated MOR in the DRt indicate that the inhibitory opioidergic control over this facilitatory area becomes compromised, likely through receptor desensitization and degradation [84]. These findings are important because they show that the loss of opioid efficacy in chronic pain may reflect, at least in part, impaired opioidergic restraint over descending facilitatory circuitry rather than a generic failure of analgesia alone.

This point becomes even more important when considering the possibility that opioidergic plasticity contributes not only to analgesic failure but also to paradoxical pain amplification. Chronic morphine exposure has been shown to shift MOR signaling in the DRt from inhibitory to excitatory, with increased phosphorylated cAMP response element-binding levels, reversal of morphine-induced hypersensitivity by local lidocaine, and attenuation of opioid-induced hyperalgesia after MOR knockdown or ultra-low-dose naloxone at the DRt [85]. These findings indicate that opioid-induced hyperalgesia should be understood not merely as a peripheral or pharmacological anomaly, but as a circuit-level disorder involving maladaptive reorganization of descending control. The DRt is particularly relevant here because it offers a concrete substrate through which altered MOR signaling may enhance descending facilitatory gain rather than simply reduce inhibitory tone. Crucially, this form of DRt plasticity may also compromise endogenous pain-inhibits-pain mechanisms. In chronic joint pain, μ-opioid receptor activation at the DRt has been shown to shift diffuse noxious inhibitory controls from analgesia to hyperalgesia, supporting the view that MOR signaling in this facilitatory nucleus can become functionally maladaptive under persistent pain conditions [86]. More broadly, a shift in μ-opioid receptor coupling from inhibitory Gi to excitatory Gs proteins within the DRt provides a plausible mechanistic framework for the loss or reversal of DNIC in chronic pain states [87]. In this maladaptive state, MOR activation no longer restrains facilitation but instead promotes it, helping explain why opioid modulation may become markedly less effective in chronic pain states. Importantly, such region-specific MOR plasticity in the DRt may be pharmacologically reversible, including through ultra-low-dose naloxone strategies that favor restoration of inhibitory signaling, thereby reinforcing the therapeutic relevance of circuit-specific opioid mechanisms in the brainstem [86,87].

A complementary example is provided by recent placebo studies showing that endogenous opioid signaling in the vlPAG causally gates placebo analgesia, further supporting the idea that opioidergic control is implemented through defined descending brainstem circuits rather than through diffuse neurochemical influence alone [79]. Opioids should therefore be interpreted at the level of circuits rather than as a global chemical influence. Reviews focusing on brainstem opioid control highlight the need to distinguish opioid actions across PAG, RVM, DRt, and related pathways, as well as to consider their effects on different neuronal subpopulations and circuit motifs [5,15]. This makes opioidergic signaling particularly suitable as a bridge between classical pain neurobiology and current circuit-level interpretation.

### 6.2. Serotonergic Signaling

At the neurochemical level, the main issue is not whether descending serotonin is globally inhibitory or facilitatory, but how spinal receptor subtype and circuit state determine its effect on nociceptive transmission [1,9,19]. Serotonergic influences associated with raphe–RVM-related systems act on multiple spinal receptor subtypes, with divergent functional consequences for dorsal horn processing [19,37,38,39]. This receptor-level heterogeneity is central to understanding why serotonergic drive can contribute to inhibition under some conditions and facilitation under others.

This issue becomes especially important in persistent pain states. In neuropathic pain, part of the shift toward descending facilitation appears to occur at the spinal receptor level, where facilitatory 5-HT3 receptor signaling in the dorsal horn becomes functionally more prominent and can bias serotonergic drive toward pronociception even without requiring large global changes in serotonin tone [2,19,37]. In this setting, the functional effect of serotonin depends less on transmitter presence alone than on which receptor populations are available, where they are expressed, and how they interact with the prevailing excitability of dorsal horn circuits.

Serotonergic signaling may therefore exacerbate hypersensitivity under some neuropathic conditions while contributing to inhibition or therapeutic efficacy under others, depending on receptor distribution, circuit context, and interaction with other descending systems such as the noradrenergic pathway [11,19,37,39]. This helps explain why pharmacological manipulation of monoaminergic systems can produce variable effects across pain states. In chronic pain, serotonergic influence may contribute not only to diminished inhibition, but also to altered gain control and maintenance of facilitatory bias when receptor-specific signaling is reorganized across the descending modulatory network [11,19,37,39].

Thus, serotonin is best treated not as a pathway label, but as a receptor-dependent modulatory system. Its contribution to descending pain control depends on spinal target, receptor subtype, and network state, making it a useful example of why neurochemical mechanisms cannot be inferred from transmitter identity alone.

### 6.3. Noradrenergic Signaling

Noradrenergic signaling, particularly through locus coeruleus–spinal projections, remains one of the best-established neurochemical substrates of endogenous descending inhibition [19,41,42,43]. Spinal noradrenaline acting at α2-adrenoceptors is classically linked to suppression of nociceptive transmission, and this basic mechanism continues to underlie the therapeutic rationale for several analgesic strategies that enhance noradrenergic tone [41,42,44]. At the same time, noradrenergic modulation should not be reduced to a uniform inhibitory effect, since receptor subtype, target circuitry, and physiological state shape its final impact on nociceptive processing [9,42,43].

This complexity is important because noradrenaline does not have a fixed functional meaning across descending circuits. Although α2-adrenoceptor-mediated inhibition remains the best-established mechanism, noradrenergic effects can vary with spinal target, receptor distribution, and the broader balance between inhibitory and facilitatory network influences [9,42,43]. In some contexts, these effects may become facilitatory. This is particularly evident in the DRt, a facilitatory pain-control area in which increased noradrenaline enhances nociceptive behavior and α1-adrenoceptor blockade attenuates inflammatory and neuropathic pain-related responses [88,89]. This reinforces the need to interpret noradrenergic signaling at the level of receptor subtype, anatomical site, and circuit state rather than as uniformly inhibitory.

This duality is best interpreted in mechanistic rather than contradictory terms. Under many conditions, recruitment of descending noradrenergic pathways contributes to antinociception and inhibitory control [41,42]. However, the protective value of that system may diminish in chronic pain, where altered recruitment, reduced efficacy, or interaction with facilitatory circuitry may change its functional significance [9,11,19,43,90]. Noradrenergic signaling may therefore become insufficient not simply because the pathway is absent, but because its inhibitory influence is no longer effectively recruited, appropriately weighted, or adequately integrated within the broader descending network [9,11]. Consistent with this view, recent ultrahigh-field human imaging shows that the LC is engaged within distinct brainstem network configurations associated with both hypoalgesic and hyperalgesic CPM responses, reinforcing the idea that LC-dependent modulation depends on network state rather than a fixed inhibitory role [31].

Recent circuit studies also support the idea that noradrenergic output is embedded within defined long-range and local brainstem pathways rather than operating as an isolated descending signal. Cortical and PAG-linked pathways can recruit downstream brainstem antinociceptive systems. Recent work has also shown that vlPAG inputs to the LC, RVM inputs to the LC, and peri-LC microcircuits shape noradrenergic contributions to pain and anxiety-related behavioral outputs [9,10,11,73,74,89]. Conceptually, this helps move discussion beyond the older idea of a single inhibitory noradrenergic tract toward a view in which noradrenergic modulation is one component of a broader and dynamically interacting system of pain control.

This state-sensitive view also has translational value. It helps explain why drugs acting on monoaminergic systems may have variable effects across pain conditions and why pharmacological enhancement of noradrenergic tone may be more beneficial in some chronic pain states than in others [11,19,42]. In that sense, noradrenaline is not simply an inhibitory transmitter, but a major neurochemical substrate through which adaptive and maladaptive reorganization of descending control acquires clinical relevance [9,11,19].

### 6.4. Cannabinoid Contributions to Endogenous Pain Modulation

Cannabinoid mechanisms are relevant to descending pain modulation and form part of the broader endogenous neurochemical architecture alongside opioidergic and monoaminergic systems [2,91,92,93]. Experimental work has shown that cannabinoid receptor activation can suppress nociceptive neuronal activity within the spinal dorsal horn, particularly through CB1 receptor-related mechanisms, and subsequent research has expanded this view into an endocannabinoid model of pain regulation extending across peripheral, spinal, and supraspinal levels [91,94,95]. This makes the cannabinoid system an important endogenous modulator of nociceptive signaling rather than merely a pharmacological add-on.

Within descending pain control, cannabinoids are especially relevant because they intersect with several of the same circuits and transmitter systems implicated in opioidergic, monoaminergic, and stress-related modulation [2,92,93,96]. This point is reinforced by evidence implicating CB1 receptors in PAG-dependent electroacupuncture antinociception and by recent findings showing that electroacupuncture exerts analgesic effects in inflammatory and neuropathic pain through CB1 receptor-mediated inhibition of GABAergic neurons in the RVM [97,98]. Together, these findings support the idea that cannabinoid mechanisms can regulate defined nodes of descending pain-control circuitry rather than acting only at spinal or peripheral sites. In this respect, cannabinoid signaling is best understood as part of the neurochemical environment that shapes how descending systems are recruited and how nociceptive signals are gated, amplified, or constrained across contexts.

Endocannabinoid mechanisms illustrate how endogenous pain modulation depends on interacting regulatory systems. Their effects are shaped by receptor localization, synaptic context, and circuit state, and may influence descending modulation by regulating synaptic efficacy, network excitability, and the balance between inhibition and facilitation [92,93,94,99]. Although the cannabinoid literature remains methodologically heterogeneous and several mechanistic details are still unresolved, the available evidence is sufficient to justify cannabinoids as part of the endogenous neurochemical architecture through which descending modulation is implemented and tuned [91,92,93,96,99].

Cannabinoid mechanisms also make clear that descending pain modulation cannot be captured by opioids and monoamines alone [1,2]. More broadly, they support a view of endogenous pain control as arising from interactions among transmitter systems rather than from a small set of classical modulators [1,92,93].

### 6.5. Neurochemical Convergence and Plasticity Across Circuits

Descending pain modulation cannot be adequately understood by treating opioids, serotonin, noradrenaline, and cannabinoids as parallel and independent channels [1,2,9,11,19]. Their significance lies instead in the way they converge within distributed circuits and how their relative influence changes across conditions. The final modulatory outcome reflects interactions among transmitters, receptors, projection-defined pathways, local microcircuits, and functional state [1,2,9,11,19]. Neurochemical signaling is therefore better understood as a set of modulatory resources recruited differently across circuit configurations and behavioral contexts.

This perspective is particularly important for chronic pain. Persistent pain states are associated not simply with altered transmitter levels, but with neurochemical reorganization embedded in circuit plasticity [11,12,15]. A given transmitter system may lose efficacy, become biased toward facilitation, or interact differently with other pathways depending on injury state, behavioral context, and prior recruitment history [11,12,15,19,90]. A particularly clear example comes from the DRt, where μ-opioid receptor activation can shift DNIC from inhibition to hyperalgesia in chronic joint pain, illustrating how the same receptor system may acquire opposite functional consequences in a different pathological context [86,87]. For this reason, neurochemical plasticity should be regarded as one dimension of network reweighting rather than as a self-contained explanatory layer [11,12,15]. The same transmitter may therefore support analgesia in one setting yet contribute to maladaptive amplification, reduced inhibitory flexibility, or treatment resistance in another.

The example of opioidergic signaling illustrates this especially well. In acute settings, opioids are strongly associated with inhibitory control and analgesia, particularly through PAG–RVM and related brainstem circuits. Yet in chronic pain and opioid-induced disorders, opioidergic signaling can become reorganized in a region-specific manner, with receptor downregulation, altered phosphorylation states, impaired inhibitory efficacy, or even a shift toward excitatory signaling in facilitatory areas such as the DRt [15,84,85]. Serotonergic and noradrenergic systems show analogous state dependence, although through different receptor and circuit mechanisms: serotonergic output varies according to receptor subtype and state, whereas noradrenergic actions depend strongly on receptor distribution, spinal target, and broader network recruitment [9,19,43]. Taken together, these examples reinforce the view that neurochemical effects are not fixed properties of transmitters themselves, but emergent properties of the circuits in which those transmitters act.

Neurochemical convergence is likely to be one mechanism through which descending systems shift across pain states. Circuit reorganization does not occur independently of chemistry, and neurochemical change does not occur independently of circuitry. Rather, transmitter systems help implement the transition from adaptive modulation to maladaptive bias by altering gain, receptor coupling, local excitability, and interactions among inhibitory and facilitatory pathways [2,9,11,19]. This is why transmitter-based explanations remain valuable, but only when interpreted within a broader circuit framework [1,2,9].

Neurochemical systems are therefore best interpreted as modulatory substrates operating within dynamic, interacting circuits. This perspective preserves the value of classical transmitter-based insights while aligning more closely with current circuit neuroscience. It also provides a useful bridge to the next section, where descending modulation is considered across different pain states and where neurochemical and circuit reorganization become inseparable [2,11,12]. The central implication is that, in descending modulation, transmitters do not carry fixed meanings; circuits give them meaning.

## 7. Descending Modulation Across Pain States

**Figure 3:**
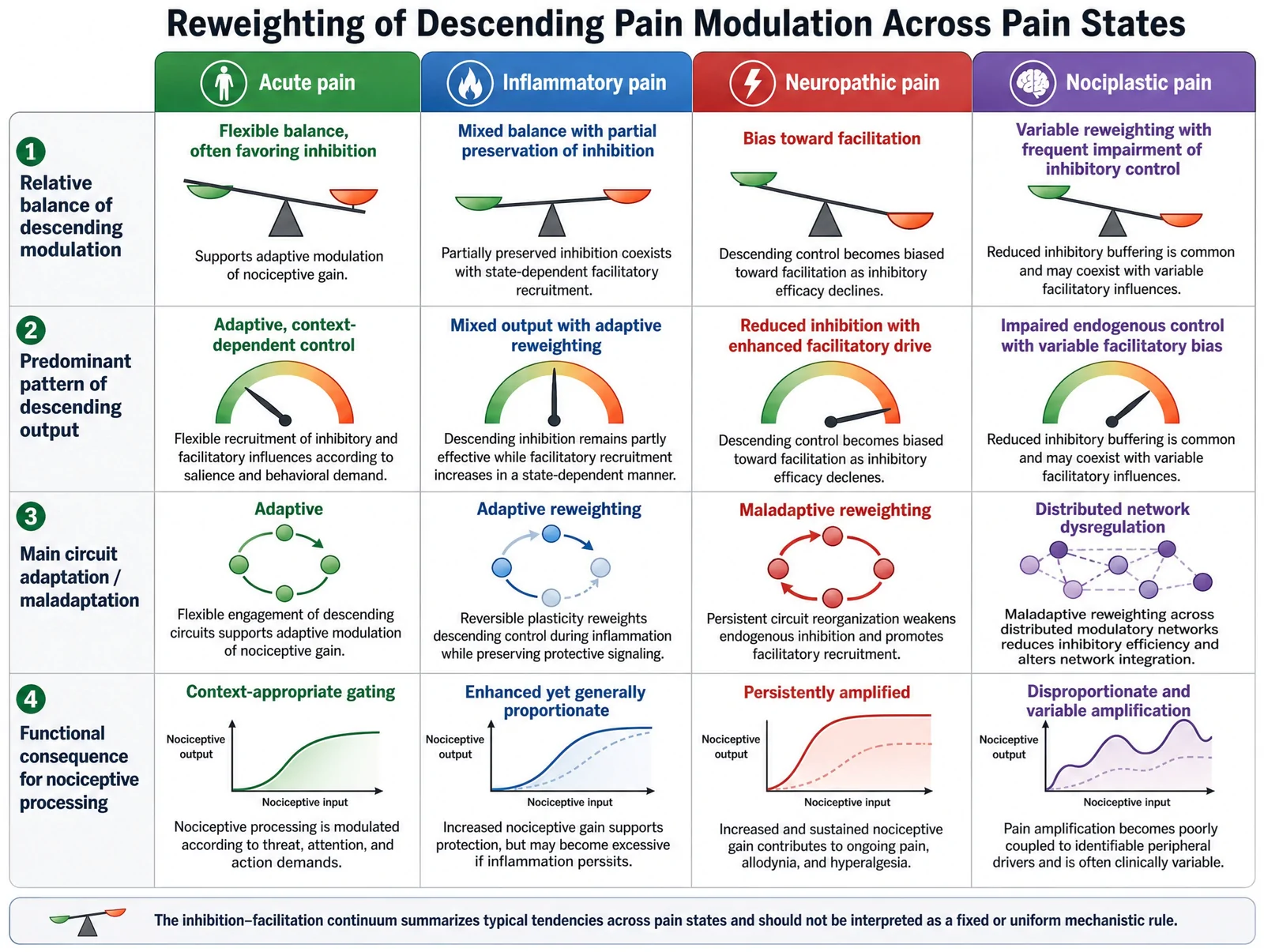
Reweighting of descending pain modulation across pain states. The figure summarizes typical changes in the relative balance, output pattern, circuit adaptation, and functional consequences of descending pain modulation across acute, inflammatory, neuropathic, and nociplastic pain states. In acute pain, inhibitory and facilitatory influences remain flexibly and contextually recruited, supporting adaptive regulation of nociceptive gain. In inflammatory pain, inhibition is partly preserved, but facilitatory mechanisms become more readily engaged as persistent peripheral input and early plasticity reweight descending output. Neuropathic pain is characterized by a more persistent facilitatory bias, reduced inhibitory efficacy, maladaptive circuit reorganization, and sustained amplification of spinal nociceptive processing. Nociplastic pain is represented as a heterogeneous state of distributed network dysregulation in which impaired endogenous control, variable facilitatory bias, and poorly calibrated nociceptive amplification may occur without a single defining peripheral lesion. The balance scales, output gauges, circuit diagrams, and input–output curves illustrate, respectively, the relative inhibitory–facilitatory weighting, predominant descending output, adaptive or maladaptive circuit reorganization, and resulting effects on nociceptive gain. The green, blue, red, and purple color coding denotes acute, inflammatory, neuropathic, and nociplastic pain, respectively. Within the circuit diagrams, arrows indicate patterns of circuit reorganization, whereas dashed curves in the input–output plots indicate the reference response. These patterns represent broad state-related tendencies rather than fixed or uniform mechanisms, since the functional sign and strength of descending modulation depend on circuit recruitment, neurochemical context, phenotype, and stage of disease. CPM, conditioned pain modulation; DNIC, diffuse noxious inhibitory controls. This state-level summary is intended to compare typical modulatory profiles across pain states rather than to depict detailed circuit connectivity. This is an author-derived conceptual schematic, not an empirically validated longitudinal model. The figure was generated with the assistance of SciFig.AI (https://scifig.ai) and ChatGPT and was subsequently reviewed, edited, and refined by the authors through successive rounds of revision to ensure scientific accuracy, conceptual coherence, and consistency with the literature.

The relative balance between descending inhibition and facilitation, the predominant pattern of modulatory output, the nature of circuit adaptation, and the resulting effects on nociceptive processing differ across acute, inflammatory, neuropathic, and nociplastic pain states. These principal state-dependent patterns are summarized in Figure 3.

### 7.1. Acute Pain: Adaptive Flexibility and Context-Dependent Control

With this integrated circuit and neurochemical framework in mind, the next question is how descending modulation is configured across different pain states. In acute pain, descending modulation calibrates nociceptive processing according to immediate behavioral priorities [2,25,100]. Under these conditions, inhibitory and facilitatory outputs are not intrinsically pathological, but serve context-dependent functions [25,26]; facilitation, in particular, may support biologically meaningful protective or recuperative behavior in acute settings [101]. Transient inhibition may allow escape, defense, focused action, or pain-induced analgesia, whereas transient facilitation may enhance the salience of recent or behaviorally relevant nociceptive input, thereby promoting protection, learning, and adaptive behavioral responses [16,25,26,101]. Acute descending control can therefore be viewed as a dynamic system that fine-tunes nociceptive gain according to environmental demands, emotional state, and ongoing behavioral needs [2,25,26].

This flexibility matters because it provides the reference point against which maladaptive changes can later be recognized. In acute conditions, modulatory balance remains sufficiently flexible to permit rapid switching between suppression and amplification of nociceptive signaling [16,25]. The key feature is not constant inhibition, but preserved adaptability [2,25]. For this reason, acute pain should not be treated merely as the earliest stage of a chronification continuum. It is also the state in which descending modulation most clearly expresses its physiological role as a behaviorally embedded regulator of nociceptive gain, rather than as a uniformly protective or uniformly harmful force [2,16,25,26].

### 7.2. Inflammatory Pain: Transitional Reorganization of Descending Balance

Inflammatory pain occupies an important intermediate position because it often preserves some adaptive modulatory features while already showing signs of circuit and neurochemical reorganization [1,2,11]. In this state, nociceptive processing is no longer regulated under the transient conditions that characterize acute pain. Persistent peripheral drive, tissue inflammation, and associated stress-related or autonomic influences can progressively alter the balance between descending inhibition and facilitation [1,11]. Rather than a simple binary transition from normal modulation to dysfunction, inflammatory pain is better conceptualized as a transitional state in which descending systems begin to be reweighted [2,11]. Inhibitory control may still be recruited, but its flexibility is often reduced, whereas facilitatory mechanisms may become more readily engaged or less effectively constrained [1,2,11,61,88].

This view helps explain why inflammatory pain can display protective and maladaptive features simultaneously. From a biological perspective, enhanced sensitivity may remain partially adaptive by promoting tissue protection and limiting further injury [1,2]. Yet the same processes may also initiate longer-lasting changes in brainstem and spinal modulation that increase the likelihood of persistent hypersensitivity [11,12]. This point is reinforced by experimental evidence from inflammatory arthritis showing that hypothalamic descending control is not simply weakened or strengthened, but reorganized in circuit-specific ways. Antinociceptive control from the paraventricular nucleus is preserved but neurochemically altered and associated with dual reorganization of RVM ON- and OFF-cell drive, whereas the normally pronociceptive influence of the dorsomedial hypothalamus on the RVM is no longer engaged in arthritic animals [102,103]. A transitional reorganization model is therefore preferable to the idea of a sharply defined threshold between physiological and pathological modulation [2,11]. It captures the fact that inflammatory pain is often the stage at which modulatory systems begin to lose contextual flexibility and move toward more persistent imbalance [1,2,11,12]. In this sense, inflammatory pain is not merely prolonged acute pain, but a state of early modulatory reorganization in which circuit and neurochemical balance may begin to shift toward more persistent maladaptive configurations.

### 7.3. Neuropathic Pain: Facilitatory Bias and Circuit Plasticity

Neuropathic pain provides one of the clearest settings in which descending modulation appears persistently reorganized [2,11,12]. Neuropathic states are frequently associated with altered engagement of brainstem pain-modulating systems, reduced inhibitory reserve, and enhanced facilitatory recruitment, although the precise balance may vary with model, time course, and phenotype [2,11,12,89,90]. This is particularly relevant for the PAG–RVM system, the locus coeruleus–spinal pathway, the dorsal reticular nucleus, and related circuit interactions, where chronic injury states are increasingly interpreted in terms of altered recruitment patterns and circuit plasticity rather than stable hard-wired output [2,11,12,69,84]. Evidence from painful diabetic neuropathy supports this interpretation, showing that the RVM exhibits increased spontaneous activity of pronociceptive ON-like cells, reduced spontaneous activity and representation of antinociceptive OFF-like cells, and an overall shift toward enhanced descending facilitation [104].

This moves the discussion beyond simplistic descriptions of neuropathic pain as either “loss of inhibition” or “gain of facilitation” [90]. In many cases, both processes likely coexist [11,90,105]. More importantly, they do so within reorganized circuits whose properties are shaped by injury-driven changes in transmitter action, receptor function, projection-defined activity, and local microcircuit coupling [11,12,89]. Recent work on defined long-range pathways reinforces the view that neuropathic pain should be understood through the lens of circuit plasticity, with descending modulation altered not only in strength but in the logic of recruitment [10,12]. The key question is not merely whether descending pathways are stronger or weaker, but how injury reshapes the conditions under which particular facilitatory or inhibitory populations are recruited. This view is also consistent with earlier work showing that descending facilitation from the rostral ventromedial medulla becomes critical for the maintenance, rather than the initiation, of neuropathic pain [105].

Experimental work on the DRt illustrates this especially well. In neuropathic models, blockade of the DRt reduces spontaneous hyperactivity, suppresses responses to heterotopic stimulation, and markedly decreases afterdischarges in spinal WDR neurons, indicating that DRt-dependent facilitation contributes to the maintenance of central sensitization rather than merely to transient amplification of nociceptive input [69]. This matters because it shows that a defined facilitatory brainstem circuit can support persistent spinal sensitization once neuropathic plasticity is established, extending earlier evidence that the DRt facilitates formalin-evoked pain behavior and enhances dorsal horn responsiveness in inflammatory conditions [61]. It also suggests that neuropathic pain is maintained not only by enhanced peripheral drive or local spinal plasticity, but also by sustained recruitment of supraspinal facilitatory loops [69].

Neuropathic pain also appears to alter the neurochemical regulation of such facilitatory circuits. At the DRt, spared nerve injury is associated with increased enkephalin levels, reduced MOR-labeled cells, and increased phosphorylated MOR, indicating that opioidergic inhibitory control over this facilitatory area becomes compromised during neuropathic pain [84]. These findings are especially relevant because they link circuit plasticity to neurochemical dysregulation: a facilitatory area may become more influential not only because it is more strongly recruited, but also because inhibitory modulation that normally restrains it becomes less effective. In that sense, neuropathic pain may reflect both enhanced recruitment of facilitation and failure of the systems that normally oppose it [84].

This formulation is also helpful translationally. It supports the idea that neuropathic pain is not a single descending phenotype, but a family of states in which descending dysfunction may differ in degree and pattern across individuals [2,11]. That is one reason why mechanism-based phenotyping remains so important and why broad statements about “descending inhibition being impaired” may be directionally correct yet mechanistically incomplete. A more informative account is that neuropathic pain reflects maladaptive reweighting within distributed descending circuits, with different combinations of facilitatory bias, reduced inhibitory flexibility, altered neurochemical control, and state-dependent recruitment across patients and models.

### 7.4. Nociplastic and Chronic Primary Pain Conditions

Nociplastic and chronic primary pain conditions present a particularly important challenge because altered pain modulation can be clinically prominent even in the absence of a single clearly identifiable lesion model [2,106]. In these conditions, disturbances in endogenous pain control are often inferred from combinations of widespread hypersensitivity, enhanced temporal summation, impaired conditioned pain modulation, and broader symptom constellations that suggest altered central processing [11,18,106]. At the same time, recent reviews caution against equating such psychophysical findings too directly with any single underlying mechanism, including “central sensitization” in a narrow technical sense [106,107]. This caution is valuable because it prevents oversimplified interpretation while still allowing descending modulation to remain a major mechanistic candidate in nociplastic pain [2,11,106].

Nociplastic pain can be viewed as a state in which distributed modulatory systems may be persistently biased toward amplification, reduced inhibitory efficiency, or impaired contextual calibration, without requiring a single anatomical lesion analogous to classical neuropathic models [2,106,107]. This makes nociplastic and chronic primary pain especially relevant to the concept of descending dysregulation as a network-level phenomenon [2,107]. It also highlights why phenotypic heterogeneity is so important: some individuals may show pronounced deficits in inhibitory control, others stronger facilitatory gain, and many likely show a mixture of altered modulation, affective loading, stress-related reorganization, and, in some cases, peripheral neuroimmune contributions, including IgG reactivity against dorsal root ganglion neurons and satellite glial cells [106,107,108,109,110].

The key point is that nociplastic pain should not be described merely as “descending inhibition failure” [2,90]. A more accurate account is that chronic primary pain states often involve reconfiguration of multiple distributed systems—including but not limited to descending pain modulation—such that nociceptive gain becomes insufficiently regulated across sensory, affective, and behavioral contexts [2,106,107]. This interpretation is consistent with recent ultrahigh-field human imaging showing spatially organized brainstem analgesic circuitry and dissociable brainstem network configurations associated with hypoalgesic and hyperalgesic conditioned pain modulation responses [22,31]. This view also fits better with current efforts toward pain stratification and mechanism-informed clinical interpretation [106,107,108].

### 7.5. Chronic Pain as Impaired Reweighting Rather than Simple Loss of Inhibition

Considered together, the patterns described across acute, inflammatory, neuropathic, and nociplastic conditions support a more nuanced account of chronic pain. The central issue is not simply that inhibitory pathways fail, nor that facilitatory pathways become uniformly dominant. Rather, chronic pain is better understood as a condition in which the capacity to flexibly reweight descending modulatory influences becomes impaired [2,11,12]. In this model, the problem is not only the sign of modulation, but also the loss of adaptive context sensitivity [2,9,12]. This interpretation is supported by recent ultrahigh-field human imaging showing that hypoalgesic and hyperalgesic CPM responses are associated with distinct brainstem network configurations rather than with uniform recruitment of a single inhibitory pathway [31]. Systems that should dynamically calibrate nociceptive gain in relation to changing behavioral demands become more rigid, more biased, or less capable of restoring balanced control [2,11,12].

This reweighting account preserves the value of classical physiological distinctions between inhibition and facilitation while avoiding reductive binary explanations. It also aligns with contemporary circuit neuroscience, which emphasizes state dependence, distributed regulation, and network plasticity across pain conditions [2,9,12]. In this sense, chronic pain may be conceptualized less as a fixed state of excessive nociceptive signaling and more as a disorder of modulatory governance, in which descending systems progressively lose their capacity for adaptive recalibration [9,11,12].

This formulation is strengthened by experimental evidence showing that chronic pain engages both facilitatory recruitment and altered neurochemical regulation within defined circuits. Rather than reflecting only a passive loss of inhibition, persistent pain can depend on the continued recruitment of supraspinal facilitatory loops that help sustain spinal sensitization. Work on the DRt illustrates this clearly: in neuropathic conditions, interference with DRt output reduces electrophysiological features of sensitization, including spontaneous neuronal hyperactivity and prolonged post-stimulus discharge, supporting the view that persistent pain is actively maintained by descending facilitatory circuitry [62,69]. Convergent work further shows that opioidergic control over this facilitatory region becomes maladaptively reorganized across chronic pain states, with impaired inhibitory restraint in neuropathic pain, excitatory MOR signaling during opioid-induced hyperalgesia, and even a shift in DNIC from analgesia to hyperalgesia in chronic joint pain [84,85,86]. These changes can be understood mechanistically through a transition in μ-opioid receptor coupling at the DRt from inhibitory Gi to excitatory Gs proteins, illustrating how a system that would normally contribute to adaptive inhibitory control may instead become biased toward facilitation in chronic states [87]. These examples support the broader view that chronic pain is sustained by maladaptive reconfiguration across circuits and transmitters rather than by a single isolated deficit.

**Figure 4:**
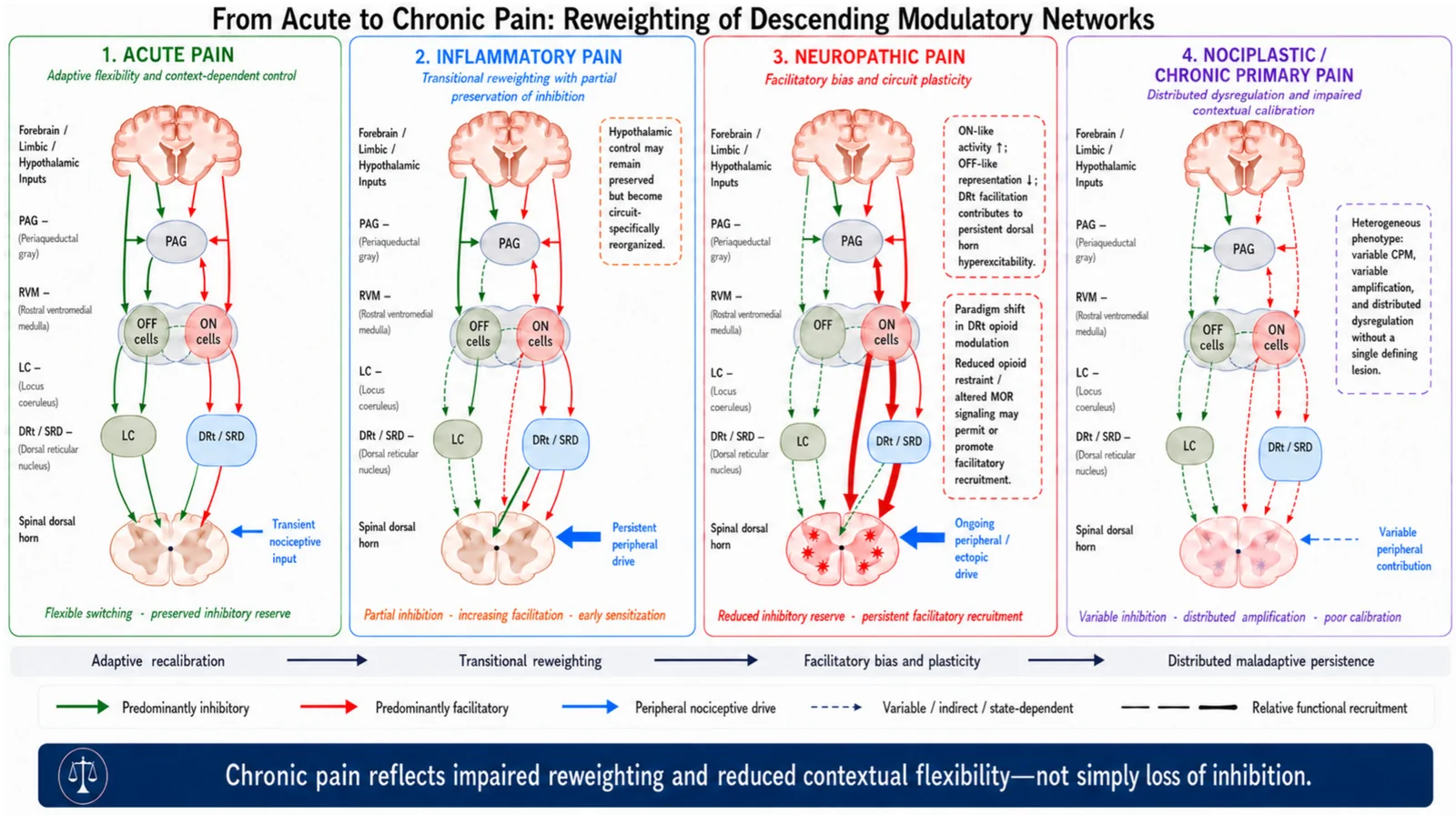
Reweighting of descending modulatory networks from acute to chronic pain states. The figure illustrates a conceptual, state-dependent reorganization of distributed descending pain-control systems across acute, inflammatory, neuropathic, and nociplastic/chronic primary pain. In acute pain, descending modulation remains flexible and context-dependent, with preserved inhibitory reserve, relatively balanced recruitment of RVM OFF- and ON-cell populations, and coordinated contributions from the periaqueductal gray (PAG), locus coeruleus (LC), dorsal reticular nucleus (DRt/SRD), and forebrain–limbic–hypothalamic inputs. The DRt/SRD is represented as supporting both ipsilateral facilitatory output and contralateral inhibitory modulation within an adaptive network configuration. In inflammatory pain, persistent peripheral nociceptive drive promotes transitional reweighting: inhibitory influences remain partly preserved, but facilitatory pathways become more readily recruited, early spinal sensitization emerges, and hypothalamic control may remain present while becoming circuit-specifically reorganized. In neuropathic pain, the balance shifts more clearly toward facilitation, with reduced OFF-like representation, increased ON-like activity, stronger DRt/SRD-related facilitatory output, ongoing peripheral or ectopic drive, and persistent spinal dorsal horn hyperexcitability. The accompanying opioid-related inset highlights a proposed paradigm shift in DRt modulation, whereby reduced opioid restraint or altered μ-opioid receptor signaling may permit or promote facilitatory recruitment rather than supporting purely inhibitory control. In nociplastic/chronic primary pain, descending modulation is depicted as a heterogeneous and distributed network disturbance, characterized by variable inhibitory impairment, inconsistent facilitatory recruitment, altered contextual calibration, and amplification that is not attributable to a single defining peripheral lesion or dominant pathway. Across panels, green arrows indicate predominantly inhibitory influences, red arrows predominantly facilitatory influences, blue arrows peripheral nociceptive drive, dashed arrows variable, indirect, or state-dependent pathways, and line thickness indicates relative functional recruitment. The overall progression emphasizes that chronic pain reflects maladaptive reweighting and reduced contextual flexibility within distributed modulatory networks, rather than a simple or uniform loss of inhibition. MOR, μ-opioid receptor; SRD, subnucleus reticularis dorsalis. In contrast to Figure 3, this circuit-level model emphasizes network reweighting across pain states and integrates brainstem, forebrain, spinal, and neurochemical components. It is an author-derived conceptual schematic, not an empirically validated longitudinal trajectory. The figure was generated with the assistance of SciFig.AI (https://scifig.ai) and ChatGPT and was subsequently reviewed, edited, and refined by the authors through successive rounds of revision to ensure scientific accuracy, conceptual coherence, and consistency with the literature.

In this account, chronic pain reflects impaired reweighting at multiple levels simultaneously: altered balance between inhibition and facilitation, altered recruitment of specific projection-defined populations, altered receptor signaling, and altered interaction between local brainstem microcircuits and broader forebrain–brainstem–spinal networks [2,9,11,12]. This is why a simple “loss of inhibition” model is often directionally correct but mechanistically incomplete [2,11,90]. It can capture one component of the syndrome while missing the active recruitment of facilitatory networks, the state-dependent failure of adaptive recalibration, and the circuit-specific neurochemical plasticity that together sustain persistent maladaptive nociceptive gain [9,12,15,90]. This model is therefore not just a semantic refinement; it provides a more accurate explanation for why chronic pain can vary across mechanisms, phenotypes, and treatment responses while still sharing common principles of dysregulated modulation [2,11,12]. The corresponding transition from adaptive modulation in acute pain to transitional reweighting in inflammatory pain, facilitatory bias and circuit plasticity in neuropathic pain, and distributed dysregulation in nociplastic or chronic primary pain is integrated at the circuit level in Figure 4.

This state-based formulation also connects naturally with human translational research. If chronic pain reflects impaired reweighting rather than any single isolated deficit, then measures such as conditioned pain modulation, temporal summation, pharmacological probing, and neuroimaging should be interpreted as partial windows onto distributed system output rather than direct readouts of a single pathway [2,11,90,108]. That perspective naturally leads into the next section, where these translational measures are considered in more detail.

## 8. Human Translational Measures of Descending Pain Modulation

### 8.1. Conditioned Pain Modulation: Utility and Limitations

CPM remains the most widely used human experimental paradigm for probing endogenous inhibitory pain control [18,108,111]. Conceptually derived from the “pain inhibits pain” phenomenon and often discussed in relation to diffuse noxious inhibitory controls (DNIC) [112], CPM is generally interpreted as a psychophysical index of the capacity of one noxious stimulus to reduce the perceived intensity of another noxious stimulus or alter the threshold response to it [18,111]. In translational terms, its major strength lies in providing a functional readout of endogenous pain inhibition in living humans without requiring invasive access to descending pathways. For that reason, CPM has become central to studies of pain sensitivity, chronic pain phenotyping, and treatment prediction [18,90,108,111,113].

At the same time, CPM should not be treated as a direct measure of a single descending pathway [20,90,114]. It reflects the integrated output of multiple interacting systems, likely including brainstem inhibitory circuits, attention, salience, autonomic state, expectancy, and broader contextual modulation [18,20,90]. Recent human imaging studies support this view by linking individual CPM variability to functional connectivity within the sgACC–PAG–RVM descending antinociceptive pathway [115]. Methodological work also underscores that CPM magnitude depends substantially on paradigm design, including the type of test stimulus, the conditioning stimulus, whether assessment is sequential or parallel, and the specific outcome measure selected [111,116,117]. Comparative studies show that these design choices can influence both sensitivity and responder classification, while recent reliability work suggests good intrasession performance under controlled conditions [117,118].

Neurophysiological and imaging studies further refine this interpretation. Work combining behavioural CPM with laser-evoked potential measures suggests that neurophysiological CPM indices may provide reproducible, brain-based markers of descending inhibition, with potential relevance for patient stratification and treatment selection [119]. At the same time, related work shows that, under matched experimental conditions, engagement of descending inhibitory control does not necessarily override spinal amplification processes such as temporal summation or wind-up [120]. In the same spirit, preclinical circuit-dissection work challenges a purely brainstem-descending interpretation of “pain inhibits pain”: using activity-dependent labeling, viral tracing, calcium imaging, and chemogenetic manipulation, Huang et al. identified an intra-somatosensory cortical S2→S1 feedforward inhibitory circuit that mediates pain-induced analgesia in mice, suggesting that cortical microcircuits may also contribute to analgesic interactions between noxious stimuli [75]. Finally, ultrahigh-field fMRI work refines this translational picture by showing that CPM in humans is associated not with a unitary “pain inhibits pain” circuit, but with distinct brainstem response profiles involving PAG, RVM, LC, SRD/DRt, and A5 depending on whether the individual expresses hypoalgesia, hyperalgesia, or no significant response [31].

A further limitation is that CPM does not map cleanly onto diagnosis or mechanism in a one-to-one manner [18,108,113]. Reduced CPM is frequently reported in chronic pain populations, yet its magnitude varies substantially within and across conditions, and not all patients with chronic pain show clear inhibitory deficits [18,90,111,113]. This heterogeneity is also evident in healthy populations, where inhibitory, absent, and facilitatory CPM profiles are associated with distinct patterns of resting-state functional connectivity across key nodes of the sgACC–PAG–RVM descending antinociceptive pathway, supporting the view that individual differences in CPM reflect network-level variation rather than a single uniform mechanism [115]. At the same time, comparative work supports the view that animal DNIC and human CPM share a common neurobiological basis within the descending inhibitory system [90,121]. In that context, recent preclinical work on DNIC suggests that impaired inhibitory control in chronic pain may, in some states, reflect loss of inhibitory opioid restraint over facilitatory nuclei such as the dorsal reticular nucleus, where a shift in μ-opioid receptor coupling from inhibitory Gi to excitatory Gs proteins can alter the net modulatory output [86,87]. Even so, the most balanced conclusion is that CPM remains a valuable translational tool whose mechanistic interpretation should remain probabilistic and network-based rather than anatomically literal [18,20,90,108,113].

### 8.2. Temporal Summation and Facilitatory Gain

Temporal summation of pain, including the progressive amplification of the delayed “second pain” component during repeated-stimulus paradigms, is commonly used as a human index of facilitatory gain within nociceptive processing [18,108,122]. It is particularly valuable because it captures a dimension of pain amplification that is conceptually distinct from CPM [108,122]. Whereas CPM is often taken as a window onto inhibitory capacity, temporal summation is generally interpreted as reflecting the tendency of nociceptive responses to build up across repeated or sustained input [18,108]. In clinical and experimental settings, this makes temporal summation especially relevant to states marked by hypersensitivity, enhanced pain amplification, or central pain facilitation [108,122].

Importantly, temporal summation should not be interpreted as the mirror image of CPM [122,123]. Findings indicate that the two measures are related but not interchangeable, and in some cohorts they show limited or no direct correlation [117,122]. This suggests that inhibitory control and facilitatory gain may vary semi-independently across individuals and conditions [108,123]. That distinction matters because it aligns with current models in which chronic pain reflects not only impaired inhibition but also altered gain control and amplified responsiveness to ongoing or repeated nociceptive input [11,108,122]. Temporal summation therefore provides an important complementary dimension to translational studies of descending modulation and pain chronification.

From a mechanistic standpoint, however, temporal summation also requires cautious interpretation. It likely reflects both spinal and supraspinal contributions, including but not limited to descending influences [11,122]. Accordingly, elevated temporal summation should not be equated automatically with a discrete facilitatory descending pathway being overactive [108,122]. Rather, it is best understood as a behavioral expression of enhanced nociceptive gain emerging from distributed nociceptive processing [11,122]. Used alongside CPM, temporal summation can help define pain modulation profiles, but neither measure should be overinterpreted in isolation [11,23,122].

### 8.3. Pharmacological Probing of Descending Systems

Pharmacological approaches provide an additional translational strategy for interrogating descending pain modulation in humans [11,19,113]. Their main value lies in linking psychophysical change to neurochemical mechanisms, particularly within monoaminergic and opioidergic systems already implicated by experimental work [11,19]. In this context, drugs that influence noradrenergic, serotonergic, or opioidergic signaling can be used not only therapeutically but also as probes of endogenous modulatory function. This approach becomes particularly valuable when psychophysical modulation profiles are linked to treatment response, as illustrated by evidence that reduced baseline CPM predicts better duloxetine efficacy in painful diabetic neuropathy [124]. More broadly, it supports the idea that endogenous pain modulation profiles may help guide mechanism-informed treatment selection in chronic pain [113]. When combined with CPM, temporal summation, or imaging readouts, pharmacological probing can therefore help relate behavioral modulation to plausible, circuit-relevant neurochemical substrates [19,20].

Yet pharmacological probing has important limits. Most systemic agents act at multiple sites and across multiple functional domains, including mood, arousal, attention, and autonomic tone, all of which can alter pain responses independently of any specific descending pathway [9,19]. In addition, the same transmitter system may contribute to inhibition in one state and facilitation in another, particularly in the case of serotonin and noradrenaline [9,11,19,88,89]. For that reason, drug response cannot be read as a clean signature of one circuit mechanism. Rather, pharmacological studies are most useful when interpreted as controlled perturbations of distributed modulatory systems whose behavioral effects depend on baseline state, receptor-specific action, and network context [9,11,19].

Pharmacological probing should therefore be understood as mechanistically informative but inherently coarse-grained. It is strongest when integrated with psychophysical and imaging measures, because convergent changes across modalities can support more disciplined inference [18,19,20]. On its own, pharmacological modulation remains an indirect method that refines rather than resolves the mechanistic interpretation of human descending pain control.

### 8.4. Human Neuroimaging and Anatomical Specificity

Human neuroimaging has become increasingly important for the translational study of descending pain modulation, especially as technical advances improve resolution within the brainstem [17,22,81]. Historically, one of the major challenges in the field was that key nuclei such as the PAG and RVM are small, deep, and physiologically noisy targets that are difficult to resolve with standard imaging approaches [17,81]. Earlier ultrahigh-field work had already shown that placebo analgesia and nocebo hyperalgesia recruit dissociable brainstem circuitry, including PAG–RVM-related pathways, supporting the feasibility of resolving functionally meaningful pain-modulatory networks in humans at 7T [125]. More recent high-field and brainstem-optimized imaging, however, has begun to provide more anatomically informative evidence that human pain modulation engages spatially structured brainstem circuitry rather than only diffuse supraspinal activity [22]. This growing anatomical resolution is especially important because it provides a translational bridge to circuit-level models in which pain-modulating nuclei such as the RVM are understood not as unitary relays, but as integrative hubs receiving both top-down and bottom-up inputs through anatomically and functionally distinct pathways [9]. In that sense, advances in ultrahigh-field and brainstem-optimized imaging are bringing human neuroimaging closer to the circuit logic of animal models, even if they still fall well short of identifying facilitatory and inhibitory neuronal populations with cellular precision.

A particularly important recent example is the demonstration of somatotopic organization within placebo-related brainstem analgesic circuitry involving the lateral PAG and RVM [22]. These data suggest that human descending modulation can show body-site-specific recruitment patterns, providing a striking bridge between animal circuit models and human functional imaging [22]. This brainstem specificity has advanced further with 7T imaging, which has identified differential PAG, RVM, LC, A5, and SRD/DRt signal changes during conditioned pain modulation, and further resolved PAG columnar contributions associated with hypoalgesic versus hyperalgesic responses [22,31]. Complementary resting-state fMRI evidence further shows that individual CPM profiles are associated with distinct functional connectivity patterns across key nodes of the sgACC–PAG–RVM descending antinociceptive pathway, reinforcing the view that variability in human pain modulation reflects network-level organization rather than a single uniform mechanism [115].

A complementary development comes from simultaneous corticospinal fMRI, which extends this translational logic beyond brainstem localization alone. Recent work identified a distributed corticospinal connectivity signature that predicts interindividual pain sensitivity, generalizes to clinical pain cohorts, and tracks rTMS-induced changes in pain perception, thereby linking experimental pain measures, patient symptoms, and neuromodulation response [77]. This does not mean that human imaging has reached the cellular precision of experimental neuroscience, but it does mark an important shift: anatomical specificity in human studies is no longer limited to broad regional labels and can now begin to address finer questions of circuit organization.

Even so, the interpretative limits remain substantial. Functional imaging signals are indirect, spatially averaged, and highly sensitive to task design, acquisition parameters, physiological-noise correction, motion, preprocessing pipelines, spatial normalization, region-of-interest definition, and analytic assumptions [17,81,114]. These issues are especially consequential in the brainstem, where small nuclei lie near cerebrospinal fluid spaces and major vessels, physiological noise is pronounced, and standard spatial smoothing or atlas-based regions of interest can blur signals across adjacent structures. Cross-study heterogeneity in field strength, pulse sequences, spatial resolution, denoising strategies, and statistical thresholds further limits direct comparison and replication. Brainstem activation therefore cannot be assumed to identify a single neuronal population, a unique modulatory sign, or a causal circuit mechanism [17,22,31]. Most human imaging evidence remains correlational; stronger causal inference will require convergent designs incorporating pharmacological perturbation, neuromodulation, lesion evidence, temporally resolved physiology, and preregistered replication. Thus, while imaging now offers stronger evidence that descending modulatory systems are anatomically organized in humans, its outputs must be integrated cautiously with animal physiology, pharmacology, and psychophysics: neuroimaging strengthens translational plausibility but does not by itself establish circuit causality [17,22,81].

### 8.5. Integrated Outputs Versus Single-Pathway Interpretations

Taken together, CPM, temporal summation, pharmacological perturbation, and neuroimaging provide a powerful translational toolkit for studying human descending pain modulation [18,19,22]. Their main strength lies in their convergence: when multiple measures point in a similar direction, confidence increases that endogenous modulatory systems are contributing meaningfully to the observed pain phenotype [19,20,22]. At the same time, none of these methods provides a direct readout of one circuit, one nucleus, or one transmitter in isolation. Each captures an integrated output shaped by multiple interacting determinants, including state dependence, task design, psychological context, and distributed network recruitment [9,18,19,20,22].

This has an important conceptual consequence. Human translational measures should not be used to force simplistic one-to-one analogies with experimentally defined pathways [11,19]. Instead, they are best interpreted as partial windows into distributed modulatory function. Such an approach is more faithful both to the strengths of modern human pain research and to the complexity of contemporary circuit neuroscience [9,22]. Descending pain modulation is not a single pathway to be measured directly, but a dynamic network whose organization and output vary across states, individuals, and clinical conditions [9,11,19,22].

## 9. Clinical and Translational Implications

### 9.1. Pain Phenotyping Through Descending Modulation Profiles

Contemporary work on descending pain modulation supports a shift from diagnosis-centered to mechanism-informed pain phenotyping [2,11,90,126]. Chronic pain conditions that appear similar at the level of symptoms or body distribution may differ substantially in their underlying modulatory organization [11,108,113]. Some patients may show relatively preserved inhibitory capacity but marked facilitatory gain, whereas others may present with more evident reduction in endogenous inhibitory control, or with mixed patterns that vary across context and disease stage [2,108,113]. This helps explain why patients carrying the same diagnostic label may differ so widely in symptom severity, sensory amplification, and treatment response [2,11,126]. Clinical clustering and ultrahigh-field imaging studies support this interpretation, showing distinct combinations of facilitatory and inhibitory modulation in young people with chronic pain and dissociable brainstem network configurations associated with different CPM response phenotypes [31,127].

A modulation-based phenotyping approach fits well with current attempts to move beyond broad syndromic categories toward more biologically grounded pain subgroups [13,107]. In this context, descending modulation should not be treated as a stand-alone explanation for all chronic pain. Rather, it represents one important dimension of a broader mechanistic profile that may also include peripheral input, central amplification, affective loading, sleep disturbance, and stress-related dysregulation [11,106,107]. Recent discussions of nociplastic pain similarly emphasize that altered nociceptive processing is clinically meaningful but heterogeneous, and that better phenotyping will require integrating psychophysical, imaging, and biological information rather than relying on symptoms alone [13,106,107].

### 9.2. Predicting Treatment Response

Descending modulation measures may also help predict therapeutic outcome [111,113,116,124,128]. This has attracted considerable attention because impaired endogenous inhibition may influence the likelihood that a patient will benefit from specific pharmacological or non-pharmacological interventions [90,108,113,124,129]. Several reviews and selected clinical studies suggest that CPM, in particular, may have predictive value in some contexts, especially when treatment mechanisms plausibly engage endogenous modulatory systems [111,113,116,123,124]. This aligns with the broader proposal that analgesic strategies should be matched to the endogenous modulatory pathways they are most likely to recruit rather than applied uniformly across heterogeneous pain phenotypes [23]. Neurophysiological CPM indices may also add value by providing reproducible markers with potential relevance for patient stratification and treatment selection [119].

At the same time, the predictive signal is not universal, and its apparent strength likely varies according to patient population, protocol design, treatment class, study context, and the reliability of the specific CPM paradigm used [111,116,128]. For this reason, the most defensible position is that descending modulation measures may contribute to treatment prediction in selected contexts, but are not yet sufficiently robust to serve as stand-alone clinical decision tools.

The appeal of treatment prediction lies in the possibility of reducing trial-and-error prescribing, particularly in chronic pain conditions where therapeutic response is often modest and highly variable. Yet the available evidence supports a probabilistic rather than deterministic use of modulation markers [20,116,128]. A patient with reduced CPM, for example, may be more likely to show a favorable response to certain treatments, as suggested by treatment-prediction studies, but such a result does not identify a single mechanism or guarantee a specific outcome [111,113,116,124]. In this sense, the clinical value of modulation-based prediction is likely to be greatest when these measures are integrated with other phenotypic information rather than interpreted in isolation [20,108,113,116].

### 9.3. Mechanism-Informed Therapeutic Strategies

A better understanding of descending pain modulation also has implications for treatment selection and therapeutic design [90,106,107,113]. If chronic pain reflects, at least in part, impaired inhibitory flexibility, enhanced facilitatory gain, or dysregulation of distributed modulatory systems, then interventions may be framed according to the processes they are most likely to recruit, restore, or rebalance [2,107,108,113]. In practice, this means asking which modulatory processes a given intervention is likely to engage, rather than applying analgesic strategies uniformly across heterogeneous pain phenotypes [23]. This applies not only to monoaminergic and opioidergic pharmacology, but also to exercise, cognitive-behavioral interventions, and other approaches capable of altering pain modulation indirectly through effects on arousal, expectation, salience, or network regulation [19,106,107,108,129,130].

At the same time, the current evidence argues against overclaiming. Mechanism-informed care is a promising direction, but it remains constrained by the incomplete specificity of available biomarkers and by the fact that most treatments engage multiple systems simultaneously [13,107,116]. Even when a therapy is thought to influence descending modulation, its effects are unlikely to be restricted to a single pathway. Accordingly, the clinical utility of a descending-modulation approach lies less in assigning one treatment to one transmitter or circuit, and more in providing a more disciplined rationale for why different patients may require different combinations or sequences of intervention [13,107,108]. This reinforces the need to view therapeutic decision-making as a stratified process in which descending modulation profiles inform—but do not determine—the selection and sequencing of interventions.

### 9.4. Heterogeneity as a Clinical Challenge and Opportunity

Patient variability is often treated as a problem in pain medicine, but from a mechanistic perspective it is also an opportunity [13,106,107]. The fact that patients differ in their descending modulation profiles means that variability should not be dismissed merely as noise; it may instead carry biologically and clinically relevant information [107,108,113]. Work on nociplastic pain, fibromyalgia, and broader chronic pain phenotypes reinforces this point by showing that altered descending control, sensory gain, and symptom burden do not cluster in the same way across all individuals [13,106,107]. In other words, heterogeneity is not just a methodological inconvenience. It is part of the phenomenon that requires explanation [13,107].

This reframing has practical consequences. It supports multidimensional assessment strategies that combine symptom-based evaluation with psychophysical testing and, where feasible, imaging or molecular markers [106,107,108,113]. Such multidimensional assessment is particularly important because descending modulation measures are influenced by inter-individual factors such as age, sex, medication use, sleep disturbance, psychological state, pain duration, pain distribution, comorbidities, and experimental paradigm characteristics. These variables should not be treated merely as sources of noise, but as potential contributors to clinically meaningful modulation profiles. It also helps explain why broad categories such as neuropathic, nociplastic, or chronic primary pain remain clinically useful yet mechanistically incomplete [106,107]. A major translational goal, therefore, is not to abolish clinical labels, but to enrich them with biologically informative dimensions, one of which is the organization of descending pain modulation [2,11,107]. Seen this way, heterogeneity becomes the rationale for phenotyping rather than an obstacle to it.

The clinical conclusion is therefore cautious but constructive. Descending pain modulation is unlikely to yield a single universal biomarker or a single-test route to personalized treatment [13,107]. However, it already provides a valuable conceptual and experimental framework for understanding inter-individual variability, refining pain phenotypes, and improving the mechanistic logic of therapeutic decision-making [2,11,107,108,113]. Its greatest clinical promise may therefore lie not in replacing existing diagnostic models, but in helping to make them more stratified, interpretable, and biologically informed [2,13,107].

## 10. Outstanding Questions and Future Directions

### 10.1. Circuit Specificity Across Pain States

If the clinical implications are increasingly clear, the mechanistic questions are far from settled. One central unresolved issue is how circuit specificity maps onto distinct pain states and how far that mapping can be made useful for clinical interpretation [2,9,11]. Descending pain modulation can no longer be reduced to a small number of invariant pathways with fixed functions [2,9]. Instead, the contribution of the PAG, RVM, locus coeruleus, DRt, and recently identified projection-defined cortical and brainstem circuits appears to depend strongly on context, injury state, behavioral demand, and time course [10,11,21].

This means that the next phase of the field will need to move beyond asking whether a circuit is “inhibitory” or “facilitatory” in general. A more important task is to define which populations are recruited in acute, inflammatory, neuropathic, and nociplastic states, under which conditions, and with what behavioral consequences [2,9,10,11,21]. Human imaging also supports this level of specificity, showing that PAG, RVM, LC, A5, and SRD do not behave as a single undifferentiated modulatory ensemble during conditioned pain modulation, but instead contribute in partially dissociable ways to inhibitory and facilitatory response phenotypes [31].

A related challenge is that anatomical regions often contain heterogeneous cell populations whose functions cannot be inferred from location alone [5,9]. The difficulty of aligning physiological, molecular, and projection-defined categories, particularly in systems such as the RVM, shows that simple regional labeling is no longer sufficient [5,9]. Future progress will depend on integrating cell-type-specific manipulation, projection-defined mapping, and state-dependent behavioral analysis, rather than treating any one of these levels as inherently primary [2,9]. In practical terms, the field now needs models that explain not only how pain is modulated, but also why the same structure may support different outputs across different states and individuals [2,5,9]. A key outstanding question is whether molecular transitions such as those identified in the DRt represent a general mechanism of circuit reweighting across descending brainstem nodes. The shift in μ-opioid receptor coupling from inhibitory Gi to excitatory Gs proteins in chronic pain and opioid-induced hyperalgesia provides one concrete example of how such state-dependent molecular plasticity might reshape descending output [85,86,87].

### 10.2. Translating Circuit-Level Animal Findings to Humans

A second major unresolved question concerns translation [2,11,17]. Animal studies now provide increasingly fine-grained accounts of descending pain circuits, but the human literature still relies largely on psychophysics, pharmacological perturbation, and neuroimaging readouts that capture integrated systems rather than single pathways [2,17]. This is not merely a technical limitation; it is also a conceptual challenge. Any attempt to bridge basic and clinical pain neuroscience must avoid assuming that a projection-defined mechanism in rodents will map neatly onto a single human biomarker or chronic pain phenotype. Translation is possible, but it depends on careful alignment of constructs, outcomes, and levels of analysis [2,11,17].

At the same time, the translational gap is narrowing. Human neuroimaging is becoming more anatomically informative, and newer work suggests that variability in conditioned pain modulation may relate to the broader functional organization of descending antinociceptive networks [18,20,22]. Even so, future translational progress will depend less on finding one perfect cross-species proxy and more on developing convergent frameworks in which animal circuit data, human imaging, and psychophysical measures are interpreted as complementary rather than interchangeable [2,20,22]. That approach is more realistic and better suited to the distributed nature of descending modulation.

### 10.3. Biomarkers and Stratification

Biomarkers of descending pain modulation remain a promising but difficult goal [13,18,20]. CPM remains the most established behavioral candidate biomarker [18,20,111,128]. Parallel efforts are therefore moving toward multimodal biomarker strategies that combine psychophysics with neuroimaging, electrophysiology, and potentially molecular or inflammatory signatures [13,20]. Reliable markers of descending pain inhibition are unlikely to emerge from behavior alone and will probably require integrated panels rather than single measures [13,20]. A further distinction is essential: group-level associations between modulation measures and pain phenotypes should not be treated as validated individual-level biomarkers. For clinical stratification, candidate markers will need to demonstrate reproducibility, predictive validity, and incremental value for individual patients, rather than only statistically significant differences between groups.

This has important implications for pain stratification. The likely future is not a single bedside test that cleanly identifies “descending inhibition failure,” but a layered phenotyping model in which modulation measures contribute one dimension of a broader pain profile [2,107]. This fits well with current work on chronic pain biomarkers and mechanism-based stratification, which emphasizes that clinically useful markers must be interpretable, reproducible, and relevant to treatment choice [2,107,116]. For descending modulation research, this means that future biomarker development should be linked not only to group differences, but also to longitudinal prediction, treatment responsiveness, and clinically meaningful subtyping [2,107,116,130].

### 10.4. Priorities for Future Research

Several priorities follow from these open questions. First, studies should be designed to compare pain states more explicitly, rather than inferring state dependence indirectly from separate studies [2,11]. The pain-state sequence used in this review should therefore be understood as a conceptual framework for organizing evidence, not as an established longitudinal trajectory through which all patients necessarily progress. Second, the field would benefit from tighter integration of circuit methods with behavioral context, so that descending modulation is studied in relation to salience, defense, action, and contextual control rather than only to reflex suppression or pain thresholds [9,12]. Third, translational studies should increasingly combine conditioned pain modulation, temporal summation, pharmacological challenge, and imaging within the same cohorts, allowing more disciplined inferences about distributed modulatory profiles [2,11]. This will be especially important for clarifying when descending inhibitory engagement can counteract ongoing spinal amplification, and when it cannot, since recent parallel human–rat work suggests that CPM-like inhibition may fail to suppress wind-up-related facilitation under some conditions [120]. Fourth, longitudinal designs will be critical for determining whether altered descending modulation precedes chronification, tracks it, or emerges as a consequence of persistent pain [2,9,12].

More broadly, future research should resist two opposite temptations: reducing descending pain modulation to one canonical pathway, or diluting it into a vague all-purpose explanation for pain variability [2,9]. The most productive path forward is to preserve mechanistic discipline while embracing the distributed, state-dependent, and multilevel nature of the system. In this sense, descending pain modulation is not merely a historical concept to be updated, but a major organizing principle through which contemporary pain neuroscience can connect circuits, behavior, biomarkers, and clinical heterogeneity. The field does not need a broader label for descending modulation; it needs more precise principles for when, where, and how descending systems reshape pain.

## 11. Conclusions

The evidence reviewed here supports a more differentiated view of descending pain modulation, in which descending control emerges from distributed, state-dependent, and neurochemically diverse circuits spanning forebrain, brainstem, and spinal levels. In this view, pain modulation is shaped not only by canonical pathways such as the PAG–RVM axis and LC–spinal projections, but also by local microcircuit interactions, the established facilitatory DRt/SRD system, and newly identified descending routes that are refining current models of nociceptive control.

Across pain states, the key issue is not merely whether inhibition is reduced or facilitation is enhanced, but how the flexible regulation of nociceptive gain becomes altered across pain states. Acute pain is characterized by adaptive contextual modulation, whereas inflammatory, neuropathic, and nociplastic states show different forms of impaired reweighting within distributed modulatory systems. In some cases, this maladaptive reweighting extends to the molecular level, as illustrated by brainstem mechanisms in which signaling systems that normally support inhibitory control become biased toward facilitation in chronic pain states. These changes have important consequences for pain persistence, heterogeneity, and treatment response. Human psychophysical, pharmacological, and neuroimaging approaches provide important but necessarily indirect windows onto this biology, reinforcing the need for cautious translational interpretation.

Revisiting descending pain modulation refines and extends classical models. The task now is to move beyond the simple recognition that descending control is bidirectional and address more precisely how distinct circuits are recruited, integrated, and reorganized across pain conditions. Ultimately, understanding when, how, and in whom descending modulation loses its adaptive flexibility may prove central not only to explaining pain chronification, but also to improving mechanistic stratification and treatment selection.

The central message is therefore clear: descending modulation is not an analgesic mechanism that sometimes fails, but a flexible control system that can be adaptively recruited, maladaptively reweighted, or pathologically reversed.

## Data Availability

No new data were created or analyzed in this study. Data sharing is not applicable to this article.

## Abbreviations

The following abbreviations are used in this manuscript:

5-HT3	5-Hydroxytryptamine receptor 3
A5	A5 noradrenergic cell group
ACC	Anterior cingulate cortex
cAMP	Cyclic adenosine monophosphate
CB1	Cannabinoid receptor type 1
CPM	Conditioned pain modulation
DNIC	Diffuse noxious inhibitory controls
DRt	Dorsal reticular nucleus
fMRI	Functional magnetic resonance imaging
GABA	Gamma-aminobutyric acid
Gi	Inhibitory G protein
Gs	Stimulatory G protein
IgG	Immunoglobulin G
LC	Locus coeruleus
M1	Primary motor cortex
mGluR5	Metabotropic glutamate receptor 5
MOR	μ-Opioid receptor
PAG	Periaqueductal gray
rTMS	Repetitive transcranial magnetic stimulation
RVM	Rostral ventromedial medulla
S1	Primary somatosensory cortex
S2	Secondary somatosensory cortex
sgACC	Subgenual anterior cingulate cortex
SRD	Subnucleus reticularis dorsalis
vlPAG	Ventrolateral periaqueductal gray
WDR	Wide-dynamic-range

## References

[1] Millan M.J. (2002). Descending Control of Pain. Prog. Neurobiol..

[2] Bannister K., Patel R., Hughes S. (2025). The Descending Modulation of Pain. Pain.

[3] Melzack R., Wall P.D. (1965). Pain Mechanisms: A New Theory: A Gate Control System Modulates Sensory Input from the Skin before It Evokes Pain Perception and Response. Science.

[4] Basbaum A.I., Fields H.L. (1984). Endogenous Pain Control Systems: Brainstem Spinal Pathways and Endorphin Circuitry. Annu. Rev. Neurosci..

[5] Heinricher M.M., Tavares I., Leith J.L., Lumb B.M. (2009). Descending Control of Nociception: Specificity, Recruitment and Plasticity. Brain Res. Rev..

[6] Fields H., Bry J., Hentall I., Zorman G. (1983). The Activity of Neurons in the Rostral Medulla of the Rat during Withdrawal from Noxious Heat. J. Neurosci..

[7] Lima D., Almeida A. (2002). The Medullary Dorsal Reticular Nucleus as a Pronociceptive Centre of the Pain Control System. Prog. Neurobiol..

[8] Martins I., Tavares I. (2017). Reticular Formation and Pain: The Past and the Future. Front. Neuroanat..

[9] De Preter C.C., Heinricher M.M. (2024). The ‘in’s and out’s’ of Descending Pain Modulation from the Rostral Ventromedial Medulla. Trends Neurosci..

[10] Wang G.-H., Hou X.-Y., Liu H.-Z., Zhou Z.-R., Lv S.-S., Yi L.-X., Li H., Zhang Y.-Q. (2025). Descending Projection Neurons in the Primary Sensorimotor Cortex Regulate Neuropathic Pain and Locomotion in Mice. Nat. Commun..

[11] Ossipov M.H., Morimura K., Porreca F. (2014). Descending Pain Modulation and Chronification of Pain. Curr. Opin. Support. Palliat. Care.

[12] Kuner R., Flor H. (2017). Structural Plasticity and Reorganisation in Chronic Pain. Nat. Rev. Neurosci..

[13] Van Strien W.W.J., Hollmann M.W. (2025). Pain Perception and Modulation: Fundamental Neurobiology and Recent Advances. Eur. J. Neurosci..

[14] McPherson K.B., Ingram S.L. (2022). Cellular and Circuit Diversity Determines the Impact of Endogenous Opioids in the Descending Pain Modulatory Pathway. Front. Syst. Neurosci..

[15] Costa A.R., Tavares I., Martins I. (2024). How Do Opioids Control Pain Circuits in the Brainstem during Opioid-Induced Disorders and in Chronic Pain? Implications for the Treatment of Chronic Pain. Pain.

[16] François A., Low S.A., Sypek E.I., Christensen A.J., Sotoudeh C., Beier K.T., Ramakrishnan C., Ritola K.D., Sharif-Naeini R., Deisseroth K. (2017). A Brainstem-Spinal Cord Inhibitory Circuit for Mechanical Pain Modulation by GABA and Enkephalins. Neuron.

[17] Tracey I., Mantyh P.W. (2007). The Cerebral Signature for Pain Perception and Its Modulation. Neuron.

[18] Yarnitsky D. (2010). Conditioned Pain Modulation (the Diffuse Noxious Inhibitory Control-like Effect): Its Relevance for Acute and Chronic Pain States. Curr. Opin. Anaesthesiol..

[19] Bannister K., Dickenson A.H. (2016). What Do Monoamines Do in Pain Modulation?. Curr. Opin. Support. Palliat. Care.

[20] Huynh V., Lütolf R., Rosner J., Luechinger R., Curt A., Kollias S., Michels L., Hubli M. (2022). Descending Pain Modulatory Efficiency in Healthy Subjects Is Related to Structure and Resting Connectivity of Brain Regions. NeuroImage.

[21] Li T., Zhou W., Ke J., Chen M., Wang Z., Hayashi L., Su X., Jia W., Huang W., Wang C.-S. (2025). A Pontine Center in Descending Pain Control. Neuron.

[22] Crawford L.S., Tinoco Mendoza F.A., Robertson R.V., Meylakh N., Macey P.M., Bannister K., Wager T.D., Macefield V.G., Keay K.A., Henderson L.A. (2025). Somatotopic Organization of Brainstem Analgesic Circuitry. Science.

[23] Bannister K., Hughes S. (2023). One Size Does Not Fit All: Towards Optimising the Therapeutic Potential of Endogenous Pain Modulatory Systems. Pain.

[24] Gebhart G.F. (2004). Descending Modulation of Pain. Neurosci. Biobehav. Rev..

[25] Fields H. (2004). State-Dependent Opioid Control of Pain. Nat. Rev. Neurosci..

[26] Fields H. (2007). Understanding How Opioids Contribute to Reward and Analgesia. Reg. Anesth. Pain Med..

[27] Lockwood S., Dickenson A.H. (2020). What Goes up Must Come down: Insights from Studies on Descending Controls Acting on Spinal Pain Processing. J. Neural Transm..

[28] De Preter C.C., Heinricher M.M. (2023). Direct and Indirect Nociceptive Input from the Trigeminal Dorsal Horn to Pain-Modulating Neurons in the Rostral Ventromedial Medulla. J. Neurosci..

[29] Bandler R., Shipley M.T. (1994). Columnar Organization in the Midbrain Periaqueductal Gray: Modules for Emotional Expression?. Trends Neurosci..

[30] Nguyen E., Grajales-Reyes J.G., Gereau R.W., Ross S.E. (2023). Cell Type-Specific Dissection of Sensory Pathways Involved in Descending Modulation. Trends Neurosci..

[31] Wake A.H., Crawford L.S., Meylakh N., Ramachandran A., Macefield V.G., Macey P.M., Wager T., Bannister K., Keay K.A., Henderson L.A. (2026). Differential Brainstem Circuits Mediating Conditioned Pain Modulation Induced Analgesia and Hyperalgesia: A Cross-Sectional, Ultra-High Field Functional Magnetic Resonance Imaging Study. J. Pain.

[32] Peng B., Jiao Y., Zhang Y., Li S., Chen S., Xu S., Gao P., Fan Y., Yu W. (2023). Bulbospinal Nociceptive ON and OFF Cells Related Neural Circuits and Transmitters. Front. Pharmacol..

[33] Fatt M.P., Zhang M.-D., Kupari J., Altınkök M., Yang Y., Hu Y., Svenningsson P., Ernfors P. (2024). Morphine-Responsive Neurons That Regulate Mechanical Antinociception. Science.

[34] Ellrich J., Ulucan C., Schnell C. (2000). Are ‘Neutral Cells’ in the Rostral Ventro-Medial Medulla Subtypes of on- and off-Cells?. Neurosci. Res..

[35] Schnell C., Ulucan C., Ellrich J. (2002). Atypical On-, off- and Neutral Cells in the Rostral Ventromedial Medulla Oblongata in Rat. Exp. Brain Res..

[36] Suzuki R., Rygh L.J., Dickenson A.H. (2004). Bad News from the Brain: Descending 5-HT Pathways That Control Spinal Pain Processing. Trends Pharmacol. Sci..

[37] Porreca F., Ossipov M.H., Gebhart G.F. (2002). Chronic Pain and Medullary Descending Facilitation. Trends Neurosci..

[38] Bannister K., Bee L.A., Dickenson A.H. (2009). Preclinical and Early Clinical Investigations Related to Monoaminergic Pain Modulation. Neurotherapeutics.

[39] Dogrul A., Ossipov M.H., Porreca F. (2009). Differential Mediation of Descending Pain Facilitation and Inhibition by Spinal 5HT-3 and 5HT-7 Receptors. Brain Res..

[40] Suzuki R., Rahman W., Hunt S.P., Dickenson A.H. (2004). Descending Facilitatory Control of Mechanically Evoked Responses Is Enhanced in Deep Dorsal Horn Neurones Following Peripheral Nerve Injury. Brain Res..

[41] Proudfit H.K. (1988). Chapter 27 Pharmacologic Evidence for the Modulation of Nociception by Noradrenergic Neurons. Progress in Brain Research.

[42] Pertovaara A. (2006). Noradrenergic Pain Modulation. Prog. Neurobiol..

[43] Llorca-Torralba M., Borges G., Neto F., Mico J.A., Berrocoso E. (2016). Noradrenergic Locus Coeruleus Pathways in Pain Modulation. Neuroscience.

[44] Yaksh T.L. (1985). Pharmacology of Spinal Adrenergic Systems Which Modulate Spinal Nociceptive Processing. Pharmacol. Biochem. Behav..

[45] Stone L.S., MacMillan L.B., Kitto K.F., Limbird L.E., Wilcox G.L. (1997). The α_2a_ Adrenergic Receptor Subtype Mediates Spinal Analgesia Evoked by α_2_ Agonists and Is Necessary for Spinal Adrenergic–Opioid Synergy. J. Neurosci..

[46] España J.C., Yasoda-Mohan A., Vanneste S. (2024). The Locus Coeruleus in Chronic Pain. Int. J. Mol. Sci..

[47] Alba-Delgado C., Borges G., Sánchez-Blázquez P., Ortega J.E., Horrillo I., Mico J.A., Meana J.J., Neto F., Berrocoso E. (2012). The Function of Alpha-2-Adrenoceptors in the Rat Locus Coeruleus Is Preserved in the Chronic Constriction Injury Model of Neuropathic Pain. Psychopharmacology.

[48] Taylor B.K., Westlund K.N. (2017). The Noradrenergic Locus Coeruleus as a Chronic Pain Generator. J. Neurosci. Res..

[49] Suárez-Pereira I., Llorca-Torralba M., Bravo L., Camarena-Delgado C., Soriano-Mas C., Berrocoso E. (2022). The Role of the Locus Coeruleus in Pain and Associated Stress-Related Disorders. Biol. Psychiatry.

[50] Almeida A., Leite-Almeida H., Tavares I. (2006). Medullary Control of Nociceptive Transmission: Reciprocal Dual Communication with the Spinal Cord. Drug Discov. Today Dis. Mech..

[51] Almeida A., Tavares I., Lima D. (1995). Projection Sites of Superficial or Deep Dorsal Horn in the Dorsal Reticular Nucleus. NeuroReport.

[52] Almeida A., Cobos A., Tavares I., Lima D. (2002). Brain Afferents to the Medullary Dorsal Reticular Nucleus: A Retrograde and Anterograde Tracing Study in the Rat. Eur. J. Neurosci..

[53] Leite-Almeida H., Valle-Fernandes A., Almeida A. (2006). Brain Projections from the Medullary Dorsal Reticular Nucleus: An Anterograde and Retrograde Tracing Study in the Rat. Neuroscience.

[54] Tavares I., Lima D. (1994). Descending Projections from the Caudal Medulla Oblongata to the Superficial or Deep Dorsal Horn of the Rat Spinal Cord. Exp. Brain Res..

[55] Villanueva L., Bouhassira D., Bing Z., Le Bars D. (1988). Convergence of Heterotopic Nociceptive Information onto Subnucleus Reticularis Dorsalis Neurons in the Rat Medulla. J. Neurophysiol..

[56] Villanueva L., Bouhassira D., Le Bars D. (1996). The Medullary Subnucleus Reticularis Dorsalis (SRD) as a Key Link in Both the Transmission and Modulation of Pain Signals. Pain.

[57] Villanueva L., Bing Z., Bouhassira D., Le Bars D. (1989). Encoding of Electrical, Thermal, and Mechanical Noxious Stimuli by Subnucleus Reticularis Dorsalis Neurons in the Rat Medulla. J. Neurophysiol..

[58] Almeida A., Tavares I., Lima D., Coimbra A. (1993). Descending Projections from the Medullary Dorsal Reticular Nucleus Make Synaptic Contacts with Spinal Cord Lamina I Cells Projecting to That Nucleus: An Electron Microscopic Tracer Study in the Rat. Neuroscience.

[59] Almeida A., Tavares I., Lima D. (2000). Reciprocal Connections between the Medullary Dorsal Reticular Nucleus and the Spinal Dorsal Horn in the Rat. Eur. J. Pain.

[60] Almeida A., Tjølsen A., Lima D., Coimbra A., Hole K. (1996). The Medullary Dorsal Reticular Nucleus Facilitates Acute Nociception in the Rat. Brain Res. Bull..

[61] Almeida A., Størkson R., Lima D., Hole K., Tjølsen A. (1999). The Medullary Dorsal Reticular Nucleus Facilitates Pain Behaviour Induced by Formalin in the Rat. Eur. J. Neurosci..

[62] Dugast C., Almeida A., Lima D. (2003). The Medullary Dorsal Reticular Nucleus Enhances the Responsiveness of Spinal Nociceptive Neurons to Peripheral Stimulation in the Rat. Eur. J. Neurosci..

[63] Soto C., Martín-Cora F.J., Leiras R., Velo P., Canedo A. (2008). Processing Noxious Information at the Subnucleus Reticularis Dorsalis (SRD) of Anesthetized Cats: Wind-up Mechanisms. Pain.

[64] Barik A., Thompson J.H., Seltzer M., Ghitani N., Chesler A.T. (2018). A Brainstem-Spinal Circuit Controlling Nocifensive Behavior. Neuron.

[65] Zhang L., Zhang Y., Zhao Z. (2005). Anterior Cingulate Cortex Contributes to the Descending Facilitatory Modulation of Pain via Dorsal Reticular Nucleus. Eur. J. Neurosci..

[66] Amorim D., Viisanen H., Wei H., Almeida A., Pertovaara A., Pinto-Ribeiro F. (2015). Galanin-Mediated Behavioural Hyperalgesia from the Dorsomedial Nucleus of the Hypothalamus Involves Two Independent Descending Pronociceptive Pathways. PLoS ONE.

[67] David-Pereira A., Sagalajev B., Wei H., Almeida A., Pertovaara A., Pinto-Ribeiro F. (2017). The Medullary Dorsal Reticular Nucleus as a Relay for Descending Pronociception Induced by the mGluR5 in the Rat Infralimbic Cortex. Neuroscience.

[68] Bouhassira D., Villanueva L., Bing Z., Le Bars D. (1992). Involvement of the Subnucleus Reticularis Dorsalis in Diffuse Noxious Inhibitory Controls in the Rat. Brain Res..

[69] Sotgiu M.L., Valente M., Storchi R., Caramenti G., Mario Biella G.E. (2008). Contribution by DRt Descending Facilitatory Pathways to Maintenance of Spinal Neuron Sensitization in Rats. Brain Res..

[70] Kuner R., Kuner T. (2021). Cellular Circuits in the Brain and Their Modulation in Acute and Chronic Pain. Physiol. Rev..

[71] Meylakh N., Crawford L.S., Mills E.P., Macefield V.G., Vickers E.R., Macey P.M., Keay K.A., Henderson L.A. (2024). Altered Corticobrainstem Connectivity during Spontaneous Fluctuations in Pain Intensity in Painful Trigeminal Neuropathy. eNeuro.

[72] Chen T., Taniguchi W., Chen Q.-Y., Tozaki-Saitoh H., Song Q., Liu R.-H., Koga K., Matsuda T., Kaito-Sugimura Y., Wang J. (2018). Top-down Descending Facilitation of Spinal Sensory Excitatory Transmission from the Anterior Cingulate Cortex. Nat. Commun..

[73] Lubejko S.T., Livrizzi G., Buczynski S.A., Patel J., Yung J.C., Yaksh T.L., Banghart M.R. (2024). Inputs to the Locus Coeruleus from the Periaqueductal Gray and Rostroventral Medulla Shape Opioid-Mediated Descending Pain Modulation. Sci. Adv..

[74] Harding E.K., Zhang Z., Yu W.L., Ferron L., Van Den Hoogen N.J., Trang T., Zamponi G.W. (2025). Locus Coeruleus Microcircuitry Processes Periaqueductal Gray Inputs into Distinct Outputs for Regulation of Pain and Anxiety. Cell Rep..

[75] Huang J.-Y., Jin Y.-X., Dong W.-Y., Zhao W., Cheng P.-K., Miao J.-H., Liu A., Wang D., Li J., Zhang Z. (2025). Intra-Somatosensory Cortical Circuits Mediating Pain-Induced Analgesia. Nat. Commun..

[76] Witney A.G., Larrivee D., Mansoor Rayegani S. (2020). From Mechanisms to Analgesia: Towards the Use of Non-Invasive Neuromodulation for Pain Relief in the Clinic. Neurostimulation and Neuromodulation in Contemporary Therapeutic Practice.

[77] Lin X.-M., Guo L.-F., Ni B., Zhang J.-Q., Zhang X.-S., Zhang L.-B., Wei Z.-X., Duan Y.-Y., Hu L., Zhang M. (2026). A Corticospinal Signature for Interindividual Pain Sensitivity. Nat. Commun..

[78] Apkarian A.V., Baliki M.N., Geha P.Y. (2009). Towards a Theory of Chronic Pain. Prog. Neurobiol..

[79] Livrizzi G., Chang-Weinberg J., Johnson D.A., Lubejko S.T., Liao J., Kimmey B.A., Dong C., Li Y., Beier K.T., Corder G. (2026). Top-down Control of the Descending Pain Modulatory System Drives Multimodal Placebo Analgesia. Neuron.

[80] Wolters L., Barenbrügge B., Löffler A., Bekrater-Bodmann R. (2026). Sensing vs. Seeing: Body Experience Rather than Mere Body Observation Is Linked to Efficiency of Descending Pain Modulation. Sci. Rep..

[81] Bingel U., Tracey I. (2008). Imaging CNS Modulation of Pain in Humans. Physiology.

[82] Bagley E.E., Ingram S.L. (2020). Endogenous Opioid Peptides in the Descending Pain Modulatory Circuit. Neuropharmacology.

[83] Lueptow L.M., Fakira A.K., Bobeck E.N. (2018). The Contribution of the Descending Pain Modulatory Pathway in Opioid Tolerance. Front. Neurosci..

[84] Costa A.R., Carvalho P., Flik G., Wilson S.P., Reguenga C., Martins I., Tavares I. (2019). Neuropathic Pain Induced Alterations in the Opioidergic Modulation of a Descending Pain Facilitatory Area of the Brain. Front. Cell. Neurosci..

[85] Costa A.R., Sousa M., Wilson S.P., Reguenga C., Teixeira-Pinto A., Tavares I., Martins I. (2020). Shift of Μ-Opioid Receptor Signaling in the Dorsal Reticular Nucleus Is Implicated in Morphine-Induced Hyperalgesia in Male Rats. Anesthesiology.

[86] Pereira-Silva R., Teixeira-Pinto A., Neto F.L., Martins I. (2024). μ-Opioid Receptor Activation at the Dorsal Reticular Nucleus Shifts Diffuse Noxious Inhibitory Controls to Hyperalgesia in Chronic Joint Pain in Male Rats. Anesthesiology.

[87] Pereira-Silva R., Neto F.L., Martins I. (2025). Diffuse Noxious Inhibitory Controls in Chronic Pain States: Insights from Pre-Clinical Studies. Int. J. Mol. Sci..

[88] Martins I., De Vries M.G., Teixeira-Pinto A., Fadel J., Wilson S.P., Westerink B.H.C., Tavares I. (2013). Noradrenaline Increases Pain Facilitation from the Brain during Inflammatory Pain. Neuropharmacology.

[89] Martins I., Carvalho P., De Vries M.G., Teixeira-Pinto A., Wilson S.P., Westerink B.H.C., Tavares I. (2015). Increased Noradrenergic Neurotransmission to a Pain Facilitatory Area of the Brain Is Implicated in Facilitation of Chronic Pain. Anesthesiology.

[90] Bannister K., Dickenson A.H. (2017). The Plasticity of Descending Controls in Pain: Translational Probing. J. Physiol..

[91] Hohmann A.G., Tsou K., Walker J.M. (1999). Cannabinoid Suppression of Noxious Heat-Evoked Activity in Wide Dynamic Range Neurons in the Lumbar Dorsal Horn of the Rat. J. Neurophysiol..

[92] Fine P.G., Rosenfeld M.J. (2013). The Endocannabinoid System, Cannabinoids, and Pain. Rambam. Maimonides Med. J..

[93] Bouchet C.A., Ingram S.L. (2020). Cannabinoids in the Descending Pain Modulatory Circuit: Role in Inflammation. Pharmacol. Ther..

[94] Pertwee R.G. (2001). Cannabinoid Receptors and Pain. Prog. Neurobiol..

[95] Starowicz K., Malek N., Przewlocka B. (2013). Cannabinoid Receptors and Pain. WIREs Membr. Transp. Signal.

[96] Palazzo E., Luongo L., Novellis V.D., Rossi F., Maione S. (2010). The Role of Cannabinoid Receptors in the Descending Modulation of Pain. Pharmaceuticals.

[97] Wilson-Poe A.R., Wiese B., Kibaly C., Lueptow L., Garcia J., Anand P., Cahill C., Morón J.A. (2021). Effects of Inflammatory Pain on CB1 Receptor in the Midbrain Periaqueductal Gray. PAIN Rep..

[98] Wan K., Xu Q., Shi Y., Cui C., Lei J., Zhang K., Yao Q., Rao Y., Zhou Z., Wu Y. (2025). Electroacupuncture Produces Analgesic Effects via Cannabinoid CB1 Receptor-Mediated GABAergic Neuronal Inhibition in the Rostral Ventromedial Medulla. Chin. Med..

[99] Qin R., Wu Y., Jin N., Xu X., Lan Y., Jing X., Li M. (2025). Research Progress Regarding Endocannabinoid System Involvement in Pain Modulation and Electroacupuncture Analgesia. Acupunct. Herb. Med..

[100] Bolles R.C., Fanselow M.S. (1980). A Perceptual-Defensive-Recuperative Model of Fear and Pain. Behav. Brain Sci..

[101] McNally G.P. (1999). Pain Facilitatory Circuits in the Mammalian Central Nervous System: Their Behavioral Significance and Role in Morphine Analgesic Tolerance. Neurosci. Biobehav. Rev..

[102] Pinto-Ribeiro F., Ansah O.B., Almeida A., Pertovaara A. (2008). Influence of Arthritis on Descending Modulation of Nociception from the Paraventricular Nucleus of the Hypothalamus. Brain Res..

[103] Pinto-Ribeiro F., Amorim D., David-Pereira A., Monteiro A.M., Costa P., Pertovaara A., Almeida A. (2013). Pronociception from the Dorsomedial Nucleus of the Hypothalamus Is Mediated by the Rostral Ventromedial Medulla in Healthy Controls but Is Absent in Arthritic Animals. Brain Res. Bull..

[104] Silva M., Amorim D., Almeida A., Tavares I., Pinto-Ribeiro F., Morgado C. (2013). Pronociceptive Changes in the Activity of Rostroventromedial Medulla (RVM) Pain Modulatory Cells in the Streptozotocin-Diabetic Rat. Brain Res. Bull..

[105] Burgess S.E., Gardell L.R., Ossipov M.H., Malan T.P., Vanderah T.W., Lai J., Porreca F. (2002). Time-Dependent Descending Facilitation from the Rostral Ventromedial Medulla Maintains, But Does Not Initiate, Neuropathic Pain. J. Neurosci..

[106] Nijs J., Lahousse A., Kapreli E., Bilika P., Saraçoğlu İ., Malfliet A., Coppieters I., De Baets L., Leysen L., Roose E. (2021). Nociplastic Pain Criteria or Recognition of Central Sensitization? Pain Phenotyping in the Past, Present and Future. J. Clin. Med..

[107] Kosek E., Clauw D., Nijs J., Baron R., Gilron I., Harris R.E., Mico J.-A., Rice A.S.C., Sterling M. (2021). Chronic Nociplastic Pain Affecting the Musculoskeletal System: Clinical Criteria and Grading System. Pain.

[108] Bannister K., Kucharczyk M., Dickenson A.H. (2017). Hopes for the Future of Pain Control. Pain Ther..

[109] Goebel A., Krock E., Gentry C., Israel M.R., Jurczak A., Urbina C.M., Sandor K., Vastani N., Maurer M., Cuhadar U. (2021). Passive Transfer of Fibromyalgia Symptoms from Patients to Mice. J. Clin. Investig..

[110] Fanton S., Menezes J., Krock E., Sandström A., Tour J., Sandor K., Jurczak A., Hunt M., Baharpoor A., Kadetoff D. (2023). Anti-Satellite Glia Cell IgG Antibodies in Fibromyalgia Patients Are Related to Symptom Severity and to Metabolite Concentrations in Thalamus and Rostral Anterior Cingulate Cortex. Brain Behav. Immun..

[111] Nir R.-R., Yarnitsky D. (2015). Conditioned Pain Modulation. Curr. Opin. Support. Palliat. Care.

[112] Le Bars D., Dickenson A.H., Besson J.-M. (1979). Diffuse Noxious Inhibitory Controls (DNIC). I. Effects on Dorsal Horn Convergent Neurones in the Rat. Pain.

[113] Yarnitsky D. (2015). Role of Endogenous Pain Modulation in Chronic Pain Mechanisms and Treatment. Pain.

[114] Nahman-Averbuch H., Timmers I. (2023). Neural Mechanisms Underlying the Conditioned Pain Modulation Response: A Narrative Review of Neuroimaging Studies. Pain.

[115] Li J.Z., Mills E.P., Osborne N.R., Cheng J.C., Sanmugananthan V.V., El-Sayed R., Besik A., Kim J.A., Bosma R.L., Rogachov A. (2025). Individual Differences in Conditioned Pain Modulation Are Associated with Functional Connectivity within the Descending Antinociceptive Pathway. Pain.

[116] Ramaswamy S., Wodehouse T. (2021). Conditioned Pain Modulation—A Comprehensive Review. Neurophysiol. Clin..

[117] Gil-Ugidos A., Rubal-Otero L., González-Villar A., Carrillo-De-la-Peña M.T. (2025). Conditioned Pain Modulation (CPM) Paradigms: Reliability and Relationship with Individual Characteristics. Pain Manag. Nurs..

[118] Gil-Ugidos A., Vázquez-Millán A., Samartin-Veiga N., Carrillo-de-la-Peña M.T. (2024). Conditioned pain modulation (CPM) paradigm type affects its sensitivity as a biomarker of fibromyalgia. Sci. Rep..

[119] Wang D., Zhang X., Ye S., Carter W., Finan P., Quigg M., Moosa S., Elias W.J., Liu C. (2026). Reliable Biomarkers of Descending Pain Inhibition: A Laser-Evoked Potential and Behavioural Study. Eur. J. Pain.

[120] Fieldwalker A., Patel R., Zhao L., Kucharczyk M.W., Mansfield M., Bannister K. (2025). A Parallel Human and Rat Investigation of the Interaction Between Descending and Spinal Modulatory Mechanisms. Eur. J. Pain.

[121] Sirucek L., Ganley R.P., Zeilhofer H.U., Schweinhardt P. (2023). Diffuse Noxious Inhibitory Controls and Conditioned Pain Modulation: A Shared Neurobiology within the Descending Pain Inhibitory System?. Pain.

[122] Arendt-Nielsen L., Morlion B., Perrot S., Dahan A., Dickenson A., Kress H.G., Wells C., Bouhassira D., Drewes A.M. (2018). Assessment and Manifestation of Central Sensitisation across Different Chronic Pain Conditions. Eur. J. Pain.

[123] Granovsky Y.G., Yarnitsky D. (2013). Personalized Pain Medicine: The Clinical Value of Psychophysical Assessment of Pain Modulation Profile. Rambam Maimonides Med. J..

[124] Yarnitsky D., Granot M., Nahman-Averbuch H., Khamaisi M., Granovsky Y. (2012). Conditioned Pain Modulation Predicts Duloxetine Efficacy in Painful Diabetic Neuropathy. Pain.

[125] Crawford L.S., Mills E.P., Hanson T., Macey P.M., Glarin R., Macefield V.G., Keay K.A., Henderson L.A. (2021). Brainstem Mechanisms of Pain Modulation: A within-Subjects 7T fMRI Study of Placebo Analgesic and Nocebo Hyperalgesic Responses. J. Neurosci..

[126] Edwards R.R., Dworkin R.H., Turk D.C., Angst M.S., Dionne R., Freeman R., Hansson P., Haroutounian S., Arendt-Nielsen L., Attal N. (2016). Patient Phenotyping in Clinical Trials of Chronic Pain Treatments: IMMPACT Recommendations. Pain.

[127] Ocay D.D., Ye D.-L., Larche C.L., Potvin S., Marchand S., Ferland C.E. (2022). Clusters of Facilitatory and Inhibitory Conditioned Pain Modulation Responses in a Large Sample of Children, Adolescents, and Young Adults with Chronic Pain. PAIN Rep..

[128] Nuwailati R., Bobos P., Drangsholt M., Curatolo M. (2022). Reliability of Conditioned Pain Modulation in Healthy Individuals and Chronic Pain Patients: A Systematic Review and Meta-Analysis. Scand. J. Pain.

[129] Wang Y., Aaron R., Attal N., Colloca L. (2025). An Update on Non-Pharmacological Interventions for Pain Relief. Cell Rep. Med..

[130] Bruneau A., Ferland C.E., Pérez-Medina-Carballo R., Somaini M., Mohamed N., Curatolo M., Ouellet J.A., Ingelmo P. (2022). Association between the Use of Quantitative Sensory Testing and Conditioned Pain Modulation and the Prescription of Medication and Interventional Procedures in Children with Chronic Pain Conditions. Children.

